# Spatiotemporal variation of nitrogen and phosphorus and its main influencing factors in Huangshui River basin

**DOI:** 10.1007/s10661-021-09067-1

**Published:** 2021-04-23

**Authors:** Biqiong Dong, Tianling Qin, Yu Wang, Yan Zhao, Shanshan Liu, Jianming Feng, Chenhao Li, Xin Zhang

**Affiliations:** 1 State Key Laboratory of Simulation and Regulation of Water Cycle in River Basin, China Institute of Water Resources and Hydropower Research, Beijing, China; 2 Yellow River Institute of Hydraulic Research, Yellow River Engineering Consulting Co., Ltd., Zhengzhou, China; 3 College of Resource Environment and Tourism, Capital Normal University, Beijing, China

**Keywords:** Ammonia nitrogen, Total phosphorus, Spatiotemporal variation, Influencing factor set, Partial least squares regression, Huangshui River basin

## Abstract

The foundation of managing excess nutrients in river is the identification of key physical processes and the control of decisive influencing factors. The existing studies seldom consider the influence of rainfall-runoff relationship and only focus on a few anthropogenic activities and natural attributes factors. To address this issue, a comprehensive set of influencing factors including rainfall-runoff relationship (represented by runoff coefficient), basic physical and chemical parameters of water quality, land use types, landscape patterns, topography, and socioeconomic development was constructed in this study. M–K test and cluster analysis were conducted to identify the temporal mutation and spatial clustering characteristics of NH_3_-N and TP in Huangshui River basin, respectively. Partial least squares regression was used to elucidate the linkages between water contaminants and the factors. As shown in the results, the temporal mutations of NH_3_-N and TP were obvious in the middle reaches, with 4 out of 7 catchments in the middle reaches have a larger number of mutations of NH_3_-N than other catchments. The cluster analysis results of NH_3_-N and TP among catchments were similar. This study also indicated that although the Huangshui River basin was located in the upper reaches of the Yellow River, the influences of rainfall-runoff relationship on spatiotemporal changes of NH_3_-N and TP in its sub-basins were limited. Only the temporal change of NH_3_-N in Jintan catchment in the upstream area was significantly affected by runoff coefficient. The indexes of proportion of water area (PWA), proportion of impervious area (PIA), and proportion of primary industry (PPI) were the top three influencing factors of temporal variation of NH_3_-N and TP for most catchments in the middle reaches. The temporal change of NH_3_-N in Jintan catchment in the upstream area was obviously affected by runoff coefficient. The spatial variation of NH_3_-N and TP were all affected by PWA and proportion of secondary industry significantly. The results of this study can provide theoretical basis and technical support for the control and management of nitrogen and phosphorus pollution in upper reaches of rivers.

**Supplementary information:**

The online version contains supplementary material available at 10.1007/s10661-021-09067-1.

## Introduction

**Fig. 1:**
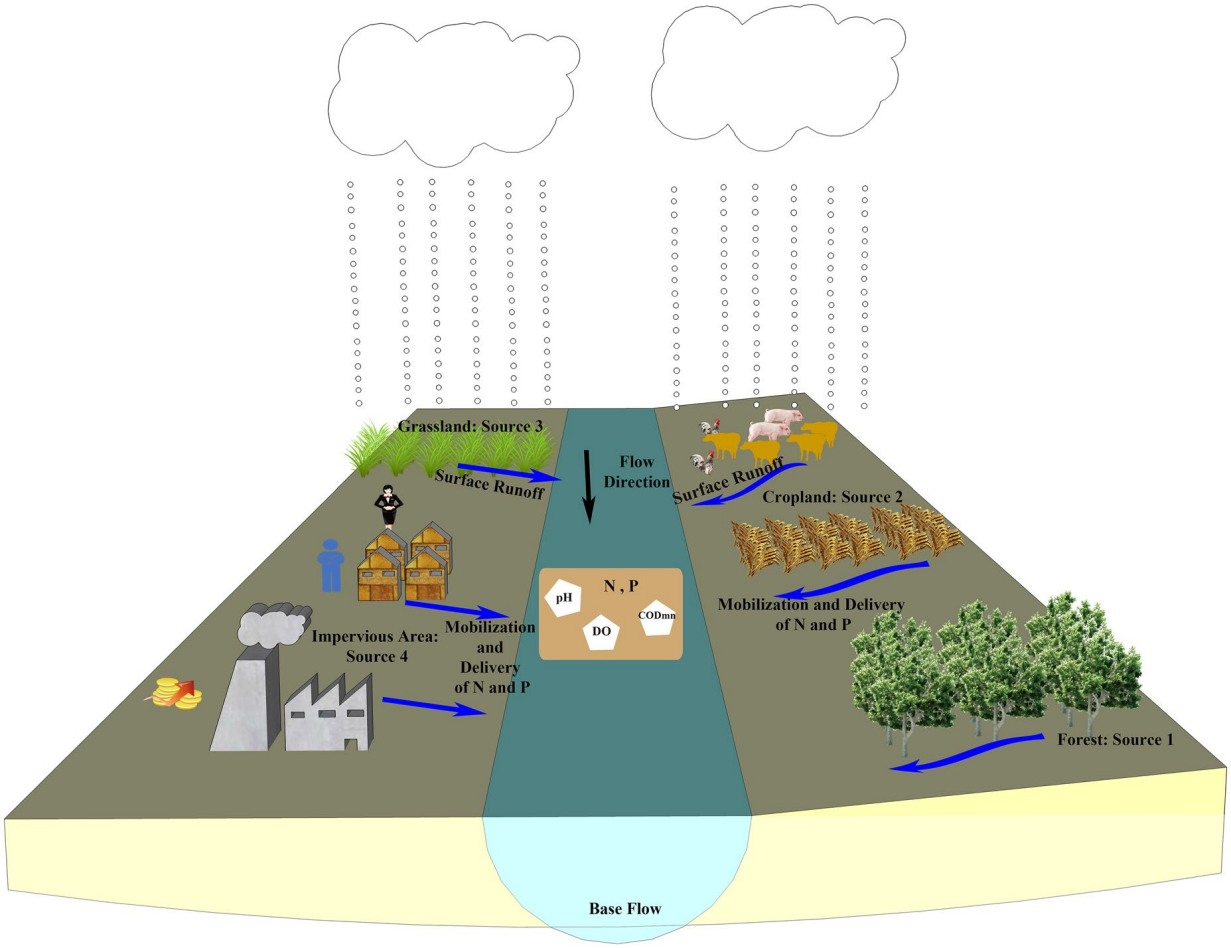
Schematic diagram of the key processes which drive the spatiotemporal variation of nitrogen and phosphorus in river

Prevention and control of river nitrogen and phosphorus pollution is one of the key issues to achieve sustainable river basin planning and management (Reichwaldt & Ghadouani, 2016; Srinivas et al., 2020). While the effectiveness of the prevention and control depends on the accurate identification of main influencing factors of pollution. Previous studies shows that the concentration of nitrogen and phosphorus in streams are regulated by a complex suite of anthropogenic activities and natural attributes (Dupas et al., 2018; Hobbie et al., 2017; Liu et al., 2018; Outram et al., 2016; Pathak et al., 2018; Pennino et al., 2016; Xia et al., 2018). However, the relative importance of different influencing factors varies in different studies especially for the upper reaches of rivers. To identify the main influencing factors which affect the spatiotemporal variation of nitrogen and phosphorus in the upper reaches of rivers, it is essential to analyze based on key physical processes. In general, the spatiotemporal variation of nitrogen and phosphorus in riverine water is driven by three key processes (Guo et al., 2019) (Fig. 1): (1) Source: the amount of the constituent within the catchment. Different land use types and socioeconomic development factors (such as the distribution of population, livestock and gross regional domestic product) are related to source and load of nitrogen and phosphorous (Li et al., 2020). (2) Mobilization: the detachment of these constituents from the source, due to weathering, erosion or biogeochemical process. The precipitation or streamflow influences the mobilization of constituents in the catchment (Mellander et al., 2015). Different land use types may cause different biogeochemical processes of nitrogen and phosphorous (Pellerin et al., 2004; Zhou et al., 2012). The basic physical and chemical parameters of water quality such as pH, DO, and COD_Mn_ influence the transformation and mobilization of nitrogen and phosphorus in rivers (Shi et al., 2016; Zhang et al., 2011). (3) Delivery: the transport of mobilized constituents from the catchment to receiving waters. The precipitation or streamflow affects the temporal variability in the delivery of the constituent to receiving waters (Guo et al., 2019). Land use types conversion significantly impacts hydrological variables, and landscape patterns affect the transport rates of allochthonous material, which both influence the transportation of nitrogen and phosphorous (Nielsen et al., 2012; Xu et al., 2019a, b). Topographic characteristics, such as elevation and slope, partly determine the transport path of pollutants from diffuse sources to rivers, especially on slope (Shi et al., 2016).

Some scholars have carried out investigations based on the identification of the abovementioned key physical processes. The impacts of basic physical and chemical parameters of water quality (Shi et al., 2016; Zhang et al., 2011), precipitation or runoff (Guo et al., 2019; Nobre et al., 2020; Shi et al., 2016), land use (Ai et al., 2015; Alvarez-Cobelas et al., 2008; Jabbar & Grote, 2019; Nobre et al., 2020; Rodrigues et al., 2018; Shi et al., 2016; Xu et al., 2019b), landscape patterns (Ai et al., 2015; Li et al., 2020; Nobre et al., 2020; Xiao et al., 2016; Xu et al., 2019a), topography (Jabbar & Grote, 2019; Shi et al., 2016), and socioeconomic development (Alvarez-Cobelas et al., 2008; Rattan et al., 2017; Wang et al., 2019) factors on the spatiotemporal variation of nitrogen or phosphorous were analyzed. However, the existing studies seldom consider the influence of rainfall-runoff relationship in the upper reaches of rivers and focus on a few aforementioned factors. There is a lack of a more comprehensive set of influencing factors which includes rainfall-runoff relationship to explore its impact on the spatiotemporal variation of nitrogen and phosphorus and to identify the key factors. Regarding the identification method of key factors, gray relational analysis (Zhang et al., 2011), redundancy analysis (Xu et al., 2019a), factor analysis (Luo et al., 2017), correlation analysis (Cui et al., 2020; Xiao et al., 2016), Bayesian networks (BNs) (Xu et al., 2019b), Bayesian hierarchical model methods (Guo et al., 2019), multiple linear regression analysis (Mayora et al., 2018; Shi et al., 2016), principal component analysis (Han et al., 2020), and partial least squares regression (Alvarez-Cobelas et al., 2008; Rattan et al., 2017) were used in various studies. The gray relational analysis method is deficient in non-standardization and non-isotonicity in the calculation of the correlation degree (Zhou, 2007). The redundancy analysis model searches for the linear combination of the independent set that maximizes the redundancy index (Oliveira et al., 2004). Factor analysis is a statistical method to extract common factors from variable groups, and correlation analysis focuses on discovering the correlation characteristics between random variables. Bayesian networks and Bayesian hierarchical model methods are suitable for large-scale data sets with vast parameters (Guo et al., 2019; Xu et al., 2019b) Although both belong to multiple linear regression analysis, partial least squares regression performs better in solving the multicollinearity problem of independent variables than Principal component analysis (Carrascal et al., 2009).

In this study, a comprehensive set of influencing factors which consisted of six types of factors including rainfall-runoff relationship (represented by runoff coefficient), basic physical and chemical parameters of water quality, land use types, landscape patterns, topography, and socioeconomic development were constructed. To test whether it is applicable to the upper reaches of rivers, we selected Huangshui River basin in the upper reaches of the Yellow River as an example. According to China Ecological and Environmental Status Bulletin from 2015 to 2018 (Ministry of Ecology and Environment of the People’s Republic of China, 2018), the main water quality pollution indexes in the Yellow River basin, NH_3_-N, and TP, were selected as the indicators of nitrogen and phosphorus pollution in this study. Based on Matlab platform, the temporal mutation characteristics and the spatial clustering characteristics of NH_3_-N and TP were obtained through M–K test and cluster analysis, respectively. Due to the interdependence of multiple factors (land use types, landscape patterns and socioeconomic development), Partial least squares regression (PLSR) was used to quantify the impacts of the influencing factor set on spatiotemporal variation of NH_3_-N and TP concentration in the Huangshui River basin from 2015 to 2018. The importance rankings of the influencing factors on NH_3_-N and TP were obtained and then the main influencing factors were acquired. The main objectives of this paper are to (1) identify temporal and spatial variation patterns of ammonia nitrogen and total phosphorus in Huangshui River basin with the available monitoring dataset and (2) determine how the temporal and spatial variation of specific water contaminant attributed to multiple factors referred in this study using PLSR. This work can provide theoretical basis and technical support for the control and management of nitrogen and phosphorus pollution in upper reaches of rivers.

## Materials and methods

### Study area

**Fig. 2:**
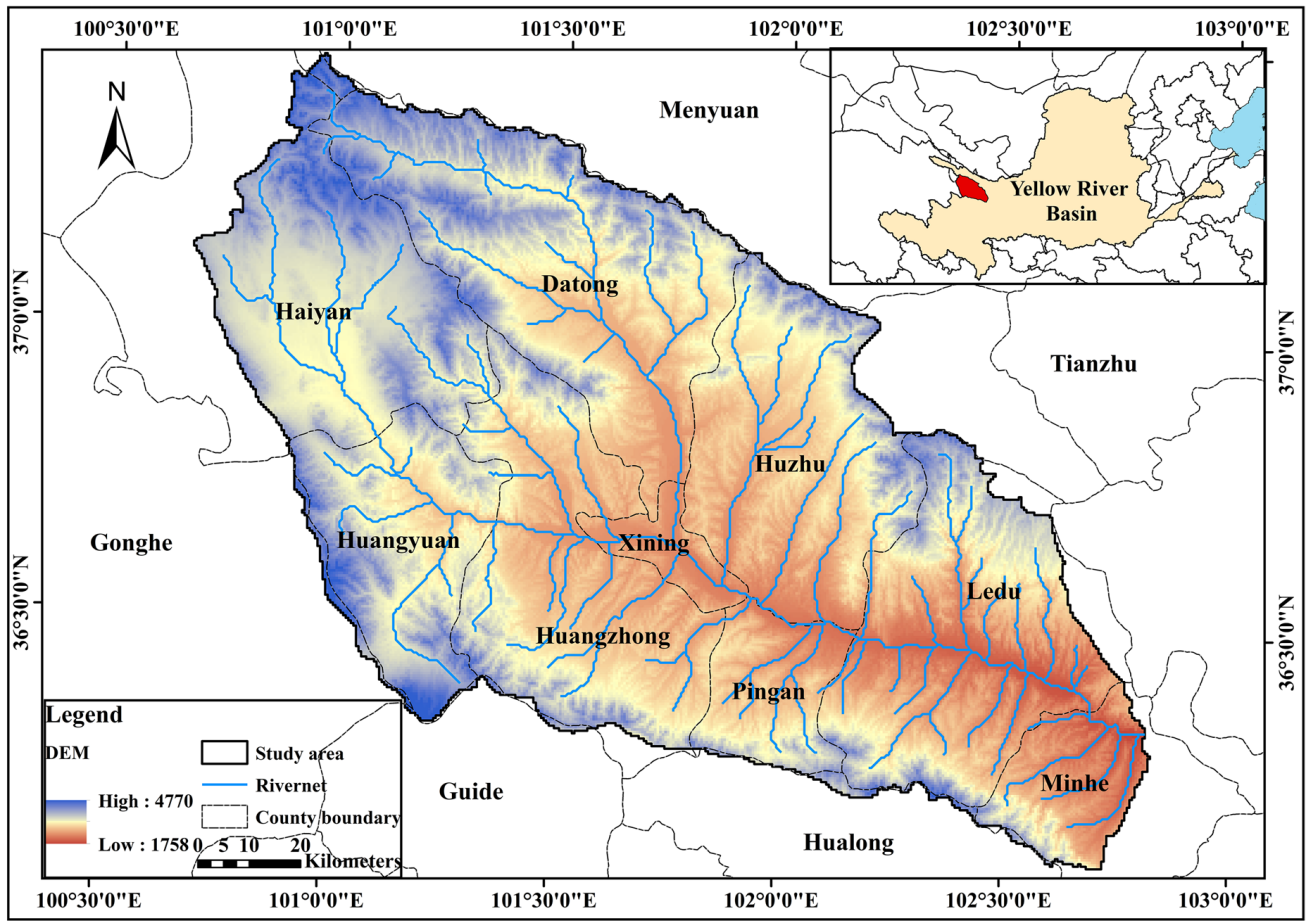
Location of Huangshui River basin

This study was conducted in Huangshui River basin, which rises in the Haibei Tibetan Autonomous Prefecture of Qinghai Province (Fig. 2). Huangshui River is a first-degree tributary of the upper reaches of the Yellow River. The river is located within 36° 02′ E–37° 8′ E and 100° 42′ N–103° 04′ N, which also lies in the transition zone between the Qinghai-Tibet Plateau and the Loess Plateau. Ecological fragility is the typical feature of this area. The total length of the main stream is 374 km, with a drainage area of 17,733 km^2^. The study site covers most of Huangshui River basin from the upper reaches of Yellow River to Minhe County, flowing through counties such as Haiyan, Huangyuan, Huangzhong, Datong, Xining, Huzhu, Ping An, Ledu, Minhe. As of 2017, the total population of the Huangshui River Basin was 3.5329 million, accounting for 60.2% of the total population of Qinghai Province (5.87 million). With the rapid development of Xining urban agglomeration in recent years, the water environment and aquatic ecology of the Huangshui River Basin have been threatened. The main stream length of this region is 278 km with a drainage area of 15,558 km^2^, as shown in Fig. 2. The terrain of the region is relative lower in the southeast and higher in the northwestern. The region has high topographic relief (elevation change of 3107 m from river source to mouth) with complex landform types (Liu et al., 2020). The region experiences an arid and semi-arid continental climate with the mean average precipitation of 381.1 mm (1960–2017 records) (Yu et al., 2015). The mean average air temperature ranged from 3.1 to 7.9 °C (1960–2017 records). The mean annual runoff at the Minhe hydrological station (the outlet of the research basin) was approximately 2053 million m^3^ (1956–2000 records). According to the land use classification results of 2017, forest was the mainly type of land use, followed by grasslands and cropland, occupying 92.91% in total.

### Materials

#### Water contamination

**Fig. 3:**
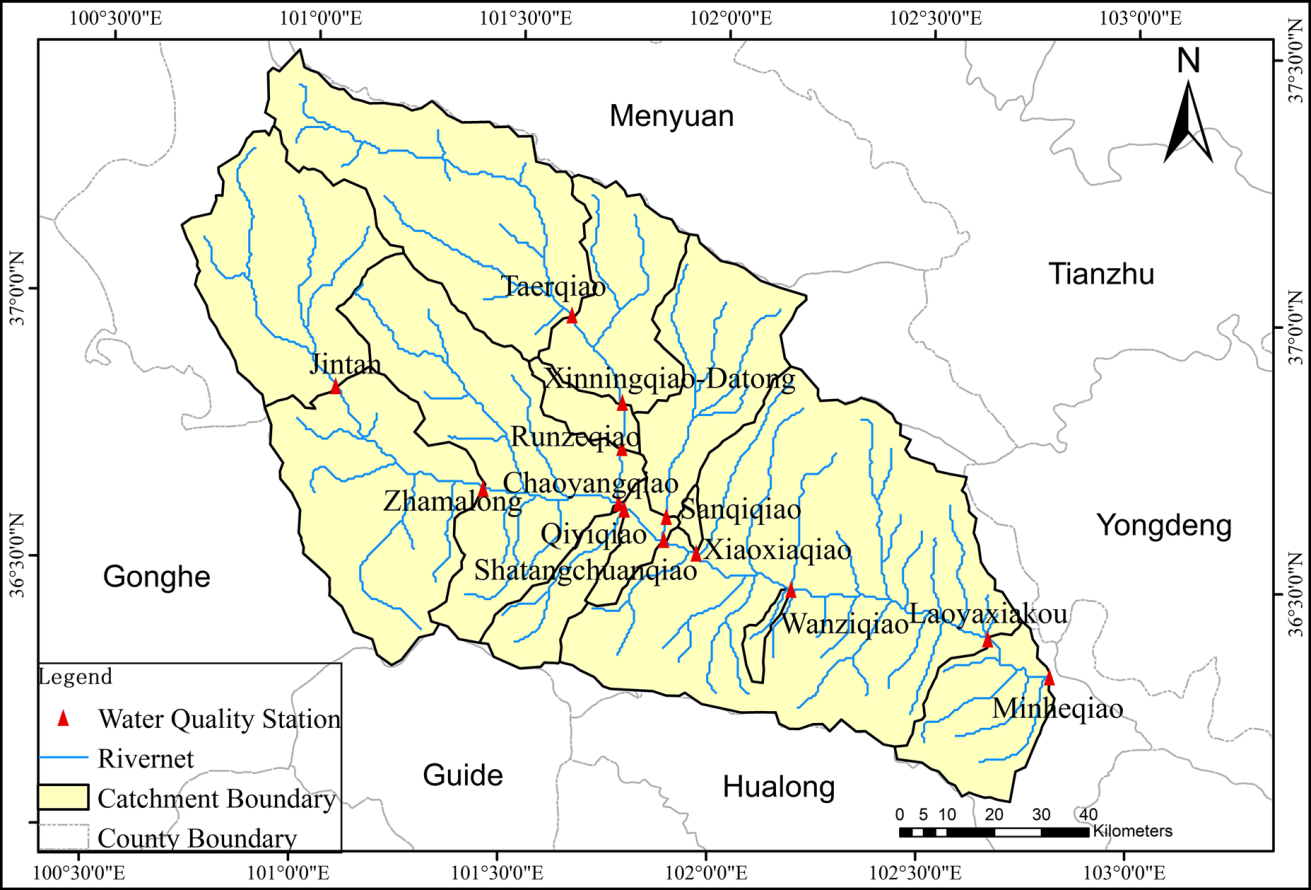
Study area and the catchments distribution based on water quality monitoring stations

We collected all water contaminant data and other spatial data from several government agencies (details in the “Water contamination,” “Potential controlling factors,” and “Spatial data” sections), statistical analysis and PLSR were combined to link them. The water contaminant dataset was supplied by the Department of Ecological Environment of Qinghai Province, China, at monthly intervals from 2015 to 2018 at 13 monitoring sites, as shown in Fig. 3. Measurements were performed on standard water samples, and all analytical techniques and calculation methods were performed according to the environmental quality standards for surface water (CSEPB, 2002). Five variables, including ammonia nitrogen (NH_3_-N), total phosphorus (TP), pondus hydrogenii (pH), the dissolved oxygen (DO), and potassium permanganate index (COD_Mn_) were analyzed in this study. The specific analysis methods for water quality items were presented as follows: NH_3_-N, spectrophotometric method with salicylic acid; TP, spectrophotometric method with ammonium molybdate; pH, glass electrode method; DO, electrochemical probe method; COD_Mn_, acidic (alkaline) potassium permanganate method.

#### Potential controlling factors

Based on previous hypothesis, the spatiotemporal variation of ammonia nitrogen and total phosphorus concentration are affected by six categories of factors. A total of 21 factors (Table 1), which belong to six aforementioned categories, were extracted as potential controlling factors in the statistical analysis. Rainfall-runoff relationship factor (that is runoff coefficient) was calculated based on the monthly outputs of rainfall and runoff from distributed hydrological model (WEP) (He et al., 2019). The land cover maps in 2015 and 2017 were used for characterizing land use type and landscape patterns were analyzed by ArcGIS 10.2 platform and the Fragstats 4.2 software, which were widely used for landscape metrics calculation (McGarigal et al., 2015). The topography data including average slope and HI were obtained by processing dem and slope data through zonal statistics module in ArcGIS 10.2. The basic data of population, livestock, regional GDP, and the added value of the primary, secondary, and tertiary industries in each district or county of the basin have three main sources. They are the National Economic and Social Development Statistical Bulletin of each district or county from 2015 to 2018, the Statistical Yearbook of each district or county from 2016 to 2019 and the China County Statistical Yearbook from 2016 to 2019. The population number, livestock quantity, regional gross product value, primary, secondary, and tertiary industries within each catchment were obtained by weighted average method. The weights were set according to the proportion of the area of each district or county in each catchment. For the convenience of comparison, livestock quantities of different species were uniformly converted into pig equivalent, and the corresponding conversion coefficients referred to the monograph of Huang et al. (2014). The values of Pop_Den, Liv_Den, PPI, PSI, and PTI in each catchment were calculated accordingly.

**Table 1:** Categories, abbreviation, and description of the selected factors in the influencing factor set

Categories	Factors	Abbreviation	Description
Rainfall-runoff relationship	Runoff coefficient (dimensionless)	RC	Annual runoff at the outlet of each catchment/annual rainfall above the outlet of each catchment
Basic physical and chemical parameters of water quality	pH	/	/
	DO	/	/
	COD_Mn_	/	/
Land use types	Proportion of forest area (2015, 2017) (dimensionless)	PFA	The ratio of forest area in a catchment
	Proportion of grassland area (dimensionless)	PGA	The ratio of grassland area in a catchment
	Proportion of water area (dimensionless)	PWA	The ratio of water area in a catchment
	Proportion of cropland area (dimensionless)	PCA	The ratio of cropland area in a catchment
	Proportion of impervious area (dimensionless)	PIA	The ratio of impervious area in a catchment
Landscape patterns	Patch density	PD	/
	Landscape shape index	LSI	/
	Patch cohesion index	COHESION	/
	Shannon’s diversity index	SHDI	/
Topography	Average slope (°)	/	Average slope gradient of a catchment
	HI	/	HI = (Hmean-Hmin)/( Hmax-Hmin) where Hmean is the mean elevation of the catchment
Socioeconomic development	Population density (person × (km^2^) − 1)	Pop_Den	Population per unit of catchment area
	Livestock density (head × (km^2^) − 1)	Liv_Den	Number of livestock per unit of catchment area
	Gross regional domestic product (ten thousand yuan)	GRDP	/
	Proportion of primary industry (dimensionless)	PPI	The ratio of the added value of the primary industry to the regional GDP in a catchment
	Proportion of secondary industry (dimensionless)	PSI	The ratio of the added value of the secondary industry to the regional GDP in a catchment
	Proportion of tertiary industry (dimensionless)	PTI	The ratio of the added value of the tertiary industry to the regional GDP in a catchment

#### Spatial data

For the basin scale, the boundary of each catchment was depicted by means of the DEM data in the ArcGIS, and each water quality monitoring station was deemed to be the outlet of the corresponding catchments (Fig. 3). Digital elevation model (DEM) data used in this study derived from National Geomatic Centre of China with resolution of 90 m*90 m. Land cover map of the basin in 2015 derived from Tsinghua University (http://data.ess.tsinghua.edu.cn/), and that in 2017 was from Department of Natural Resources of Qinghai Province, China.

### Methods

**Fig. 4:**
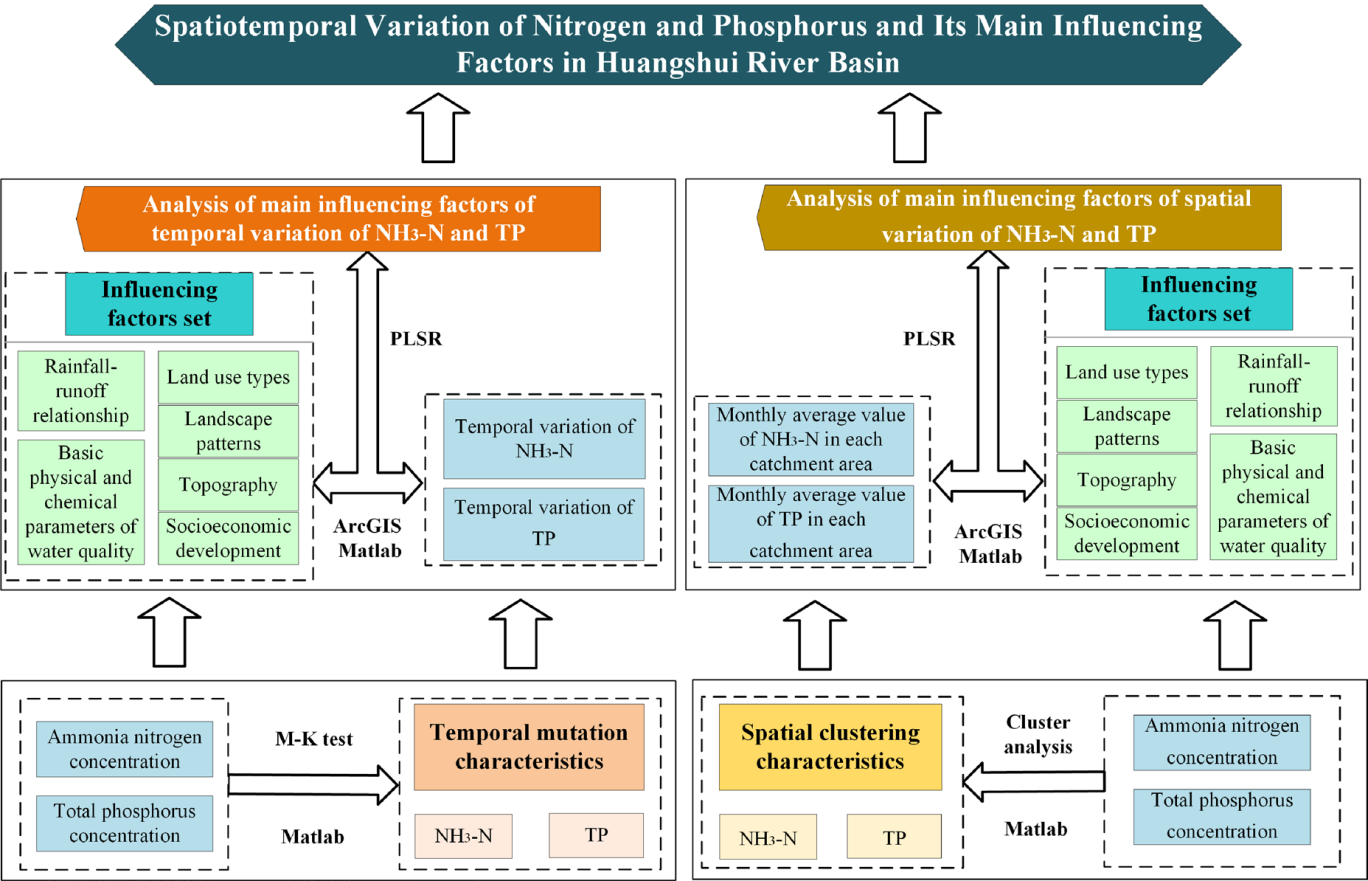
Research workflow of spatiotemporal variation of nitrogen and phosphorus concentration in Huangshui River basin and identification of main controlling factors

In this paper, an influencing factor set which includes the rainfall-runoff relationship, basic physical and chemical parameters of water quality, land use types, landscape patterns, topography, and socioeconomic development was constructed. Based on Matlab algorithms, the temporal mutation characteristics of NH_3_-N and TP in each catchment were obtained through M–K test. Moreover, the spatial clustering characteristics of NH_3_-N and TP in catchments were obtained by cluster analysis. On this basis, PLSR was used to quantify the complex interdependencies between the influencing factor set, and spatiotemporal variation of ammonia nitrogen and total phosphorus concentration in the basin. From the perspectives of temporal and spatial variation, the importance rankings of the influencing factors on NH_3_-N and TP were obtained and the main controlling factors were acquired accordingly, which lay the foundation for the accurate attribution of the temporal and spatial variation of NH_3_-N and TP (Fig. 4).

#### M–K test

The Mann–Kendall test, which is a robust and non-parametric test method, was used to diagnose the temporal trends of water quality parameters (Buendia et al., 2016; Fathian et al., 2016; Li et al., 2018). In this study, it was applied to analyze the temporal mutation characteristics of concentration of NH_3_-N and TP at each monitoring station through Matlab algorithm. Through UF statistics and UB statistics, trend and mutation time of the variable can be obtained. If UF statistics >0 (<0), the sequence of NH_3_-N or TP is on the rise (down) trend, when it exceeds the critical line (*p* < 0.05), it indicates the rise or fall of the trend is significant.

#### Cluster analysis

CA is a reduction statistical method which is commonly used to classify objects into groups according to their nearness. The objects are categorized by linking inter-sample commonalities so that the outcome demonstrates the general similarity of the components in a given set of data (Razmkhah et al., 2010). The most common approach is hierarchical agglomerative cluster analysis, which build the solution by initially assigning each document to its own cluster and then repeatedly selecting and merging pairs of clusters, to obtain a single all-inclusive cluster (Al-Murairi et al., 2014; Zhao & Karypis, 2005). The output of this cluster process is a dendrogram, and the similarities and dissimilarities are quantified through Euclidean distance measurement. In this study, hierarchical agglomerative cluster analysis was carried out to ascertain the multivariate similarity between different water quality monitoring stations based on the time series of concentration of NH_3_-N and TP in the Huangshui River basin. The CA was performed through programming and calculation on Matlab platform.

#### PLSR

The PLSR is an extension of multiple regression analysis which the effects of linear combinations of several predictors on a response variable (or multiple response variables) are analyzed. PLSR was developed for econometric modeling of multivariate time series and later was applied in chemometrics (Wold, 1995) and hyperspectral remote sensing (Min & Lee, 2005). PLSR can handle highly correlated variables on the principle of variable compression and is particularly suitable for cases in which the number of observations is less than the possible variables (Carrascal et al., 2009; Luedeling & Gassner, 2012). Mathematically, PLSR is achieved by maximizing the covariance between y and all possible linear functions of x. It is often called as the “better PCA” as it often gives improved prediction results (Luedeling & Gassner, 2012).

A PLSR model for ammonia nitrogen (NH_3_-N) and total phosphorus (TP) was established respectively to identify the main influencing factors temporally and spatially in order to obtain the attribution of spatiotemporal variation of NH_3_-N and TP. Before PLSR analyses, predictors were centered and scaled to unit variance to give them the same relative importance. The percentage of variance explained for the response variables, the cross-validated root mean squared error (RMSE), which is the difference between the predicted and observed values of each individual pass. The predictor coefficients (magnitude and direction) were used to examine the influence of predictors on responses. The PLSR was performed using Matlab algorithm.

## Results

### Temporal mutation characteristics of NH_3_-N and TP

**Fig. 5:**
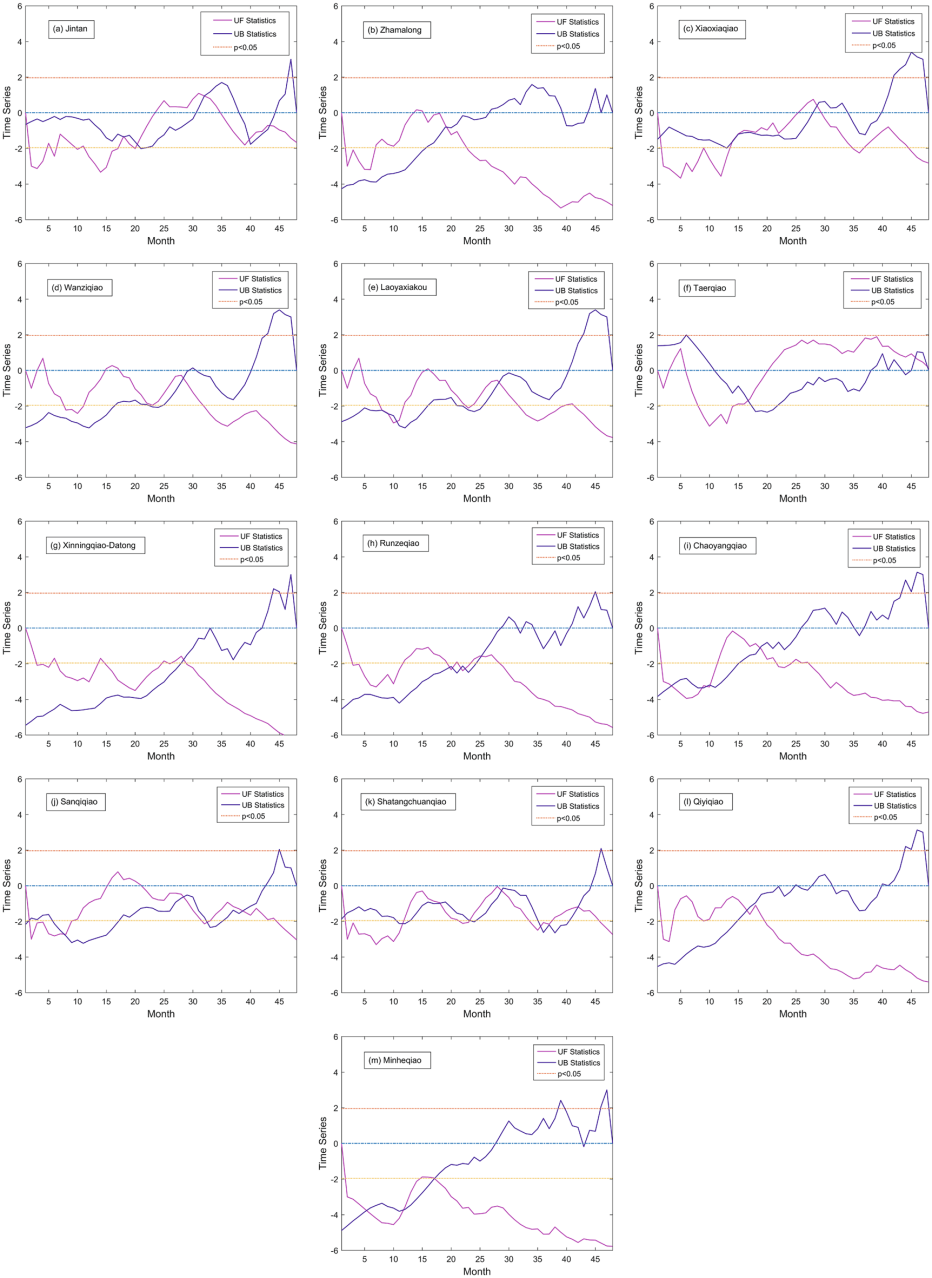
Temporal mutation characteristics of NH_3_-N in each catchment based on M–K test

**Fig. 6:**
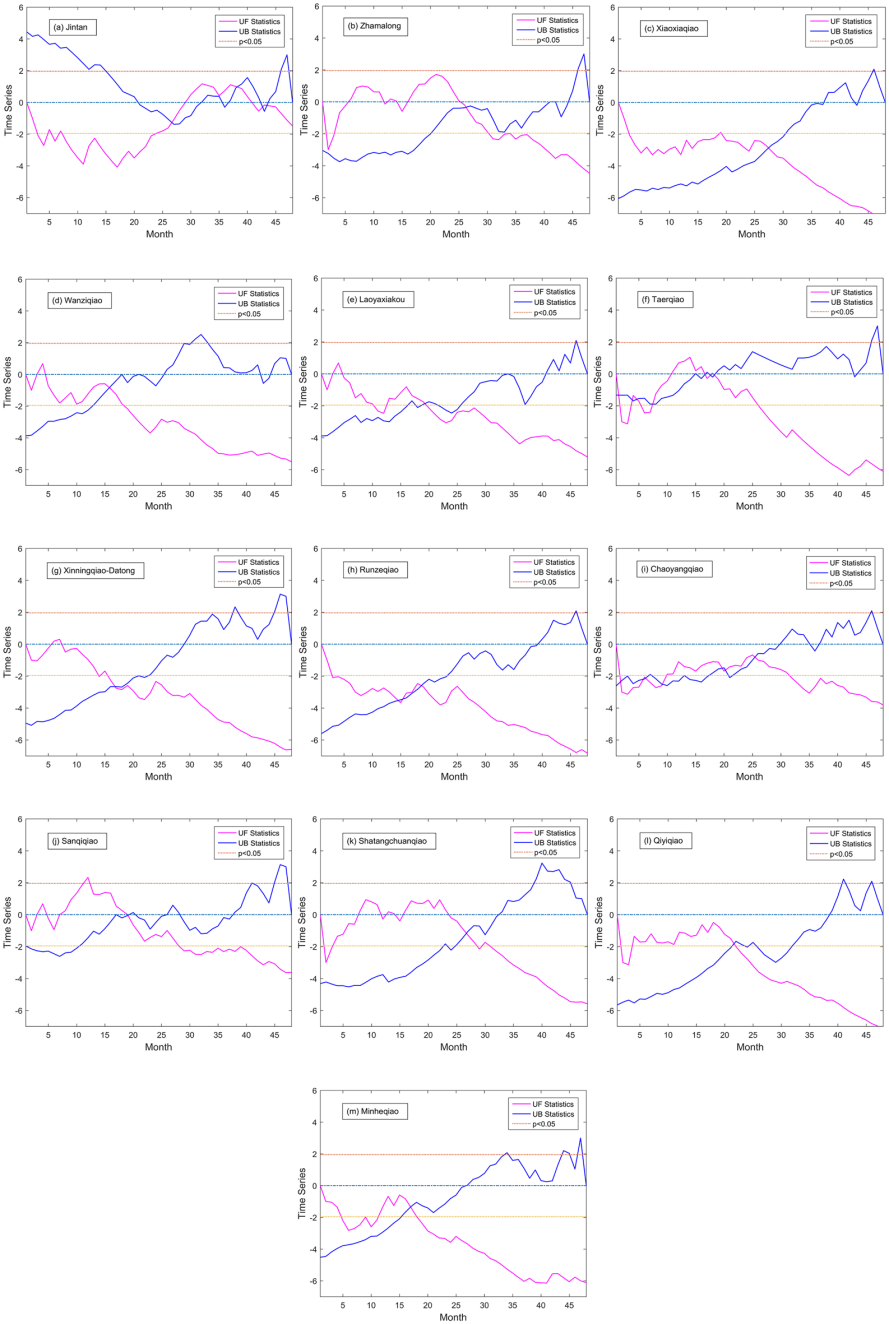
Temporal mutation characteristics of TP in each catchment based on M–K test

Less than a quarter of the 13 water quality monitoring stations showed a significant decline in the concentration of NH_3_-N and TP. As shown in Figs. 5 and 6, Xinningqiao-Datong, Minheqiao catchment showed a significant decline of NH_3_-N concentration and Runzeqiao, Xiaoxiaqiao, and Minheqiao exhibited a significant decline of TP concentration. As listed in Table S1 (Supplement Materials), the number of mutations of NH_3_-N concentration in the middle reaches of the Huangshui River basin was generally higher than that in the upper and lower reaches. Specifically, the numbers of mutations of NH_3_-N in Runzeqiao, Chaoyangqiao, Sanqiqiao, and Shatangchuanqiao catchments were all larger than 4. The numbers of mutations of NH_3_-N of the catchments in the upstream section except Jintan were less than 3, and the numbers of mutations of NH_3_-N in catchments of the downstream section were all less than 3. The numbers of mutations of TP in 11 catchments out of 13 catchments in the basin were less than that of NH_3_-N. Specifically, in the middle reaches, the numbers of mutations of TP in Runzeqiao and Chaoyangqiao catchments were equal or larger than 3. Moreover, in the upper reaches, the numbers of mutations of TP in Jintan and Taerqiao catchments were equal or larger than 3. However, the numbers of mutations of TP in catchments of the lower reaches were less.

### Spatial cluster characteristics

**Fig. 7:**
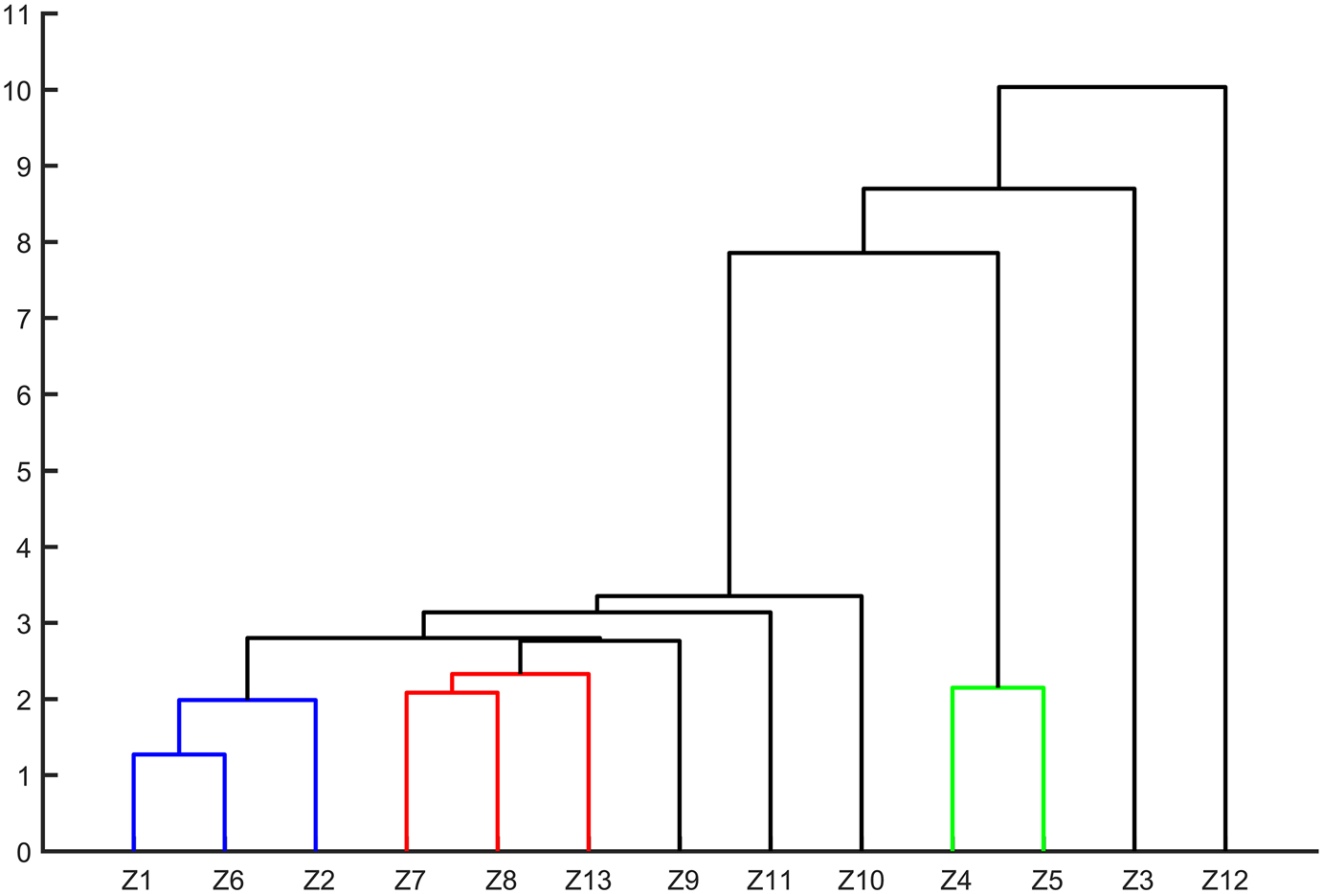
Dendrogram of cluster analysis of NH_3_-N at different water quality monitoring stations. Z1–Z13 represent the number of catchments controlled by the water quality monitoring station

The results of cluster analysis of NH_3_-N showed that Jintan, Zhamalong, and Taerqiao catchments at the upstream section belonged to entire qualified class (subject to Class III standards for surface water bodies in GB3838-2002) (CSEPB, 2002). Xinningqiao-Datong, Runzeqiao, and Minheqiao catchments belonged to basic compliance class. Wanziqiao and Laoyaxiakou catchments belonged to basic excess class. Other catchments belonged to the different categories, respectively. All of them were sectional excess categories, with the different length of the excessing period (Fig. 7, Table 2).

**Table 2:** Summary of cluster analysis results of NH_3_-N in each catchment

Serial number	Abbreviation of catchments	Category	Name of the catchment
Group.1	Z1, Z6, Z2	Entire qualified class	Jintan, Taerqiao, Zhamalong
Group.2	Z7, Z8, Z13	Basic compliance class	Xinningqiao-Datong, Runzeqiao, Minheqiao
Group.3	Z9	Sectional excess class	Chaoyangqiao
Group.4	Z11	Sectional excess class	Shatangchuanqiao
Group.5	Z10	Sectional excess class	Sanqiqiao
Group.6	Z4, Z5	Basic excess class	Wanziqiao, Laoyaxiakou
Group.7	Z3	Sectional excess class	Xiaoxiaqiao
Group.8	Z12	Sectional excess class	Qiyiqiao

**Fig. 8:**
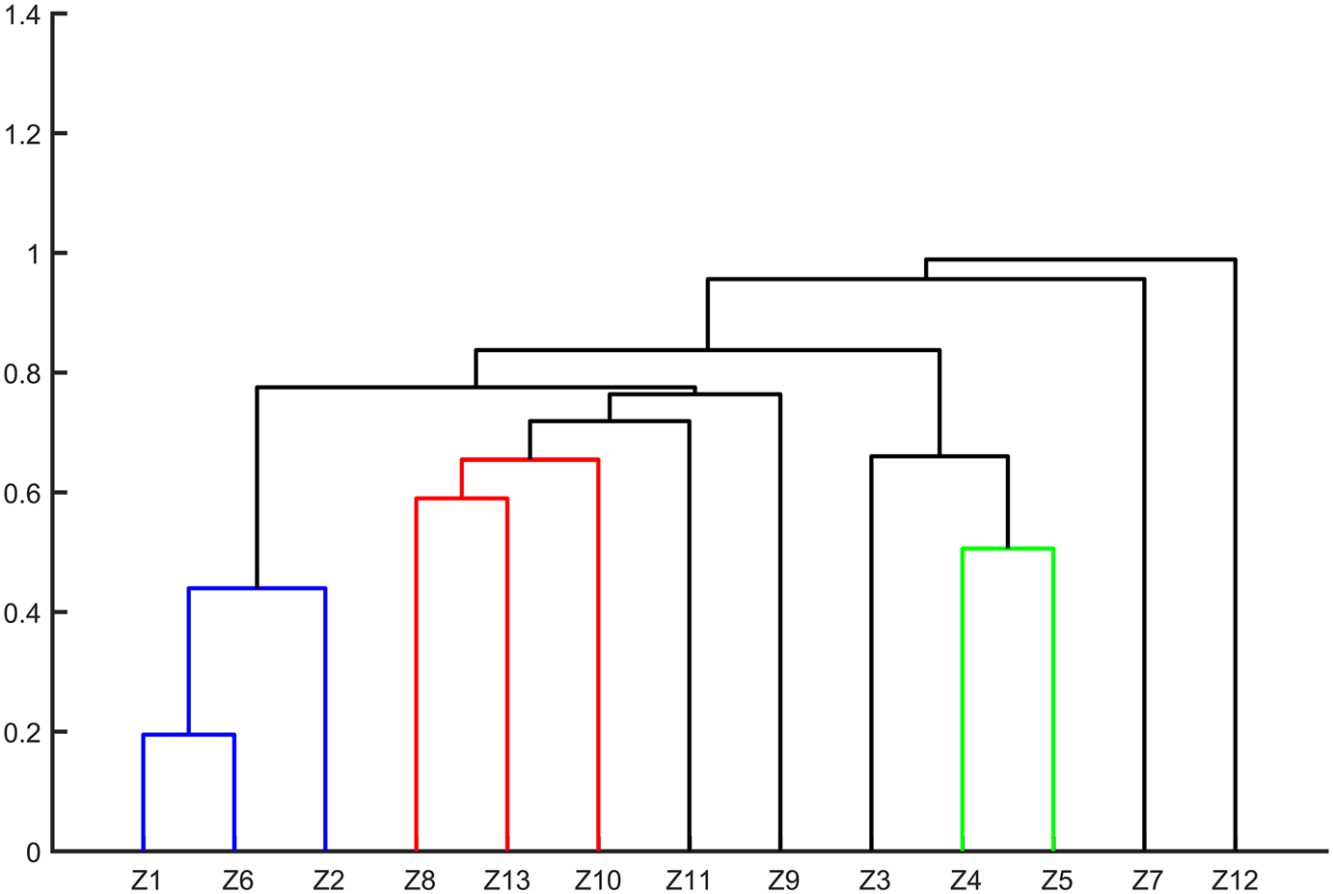
Dendrogram of cluster analysis of TP at different water quality monitoring stations. Z1–Z13 represent the number of catchments controlled by the water quality monitoring station

The results of cluster analysis of TP showed that Jintan, Zhamalong, and Taerqiao catchment at the upstream section belonged to nearly entire qualified class (subject to Class III standards for surface water bodies in GB3838-2002) (CSEPB, 2002). Runzeqiao, Sanqiqiao and Minheqiao catchments belonged to basic compliance class. Wanziqiao and Laoyaxiakou catchments fall into sectional excess class. Xiaoxiaqiao catchment pertain to basic excess class. Other catchments belonged to the different categories respectively, all of which were sectional excess categories, with the different length of the excessing period (Fig. 8, Table 3).

**Table 3:** Summary of cluster analysis results of TP in each catchment

Serial number	Abbreviation of catchments	Category	Name of the catchment
Group.1	Z1,Z6,Z2	Nearly entire qualified class	Jintan, Taerqiao, Zhamalong
Group.2	Z8,Z13,Z10	Basic compliance class	Runzeqiao, Minheqiao, Sanqiqiao
Group.3	Z9	Sectional excess class	Chaoyangqiao
Group.4	Z11	Sectional excess class	Shatangchuanqiao
Group.5	Z7	Sectional excess class	Xinningqiao-Datong
Group.6	Z4,Z5	Sectional excess class	Wanziqiao, Laoyaxiakou
Group.7	Z3	Basic excess class	Xiaoxiaqiao
Group.8	Z12	Sectional excess class	Qiyiqiao

In comparison, the cluster analysis results of NH_3_-N and TP of each catchment were similar.

There were slightly differences in the basic compliance class of the two (Group 2). The basic excess class of NH_3_-N was different from the sectional excess class of TP in Group.5. This may be due to the differences in the types of pollutants and the characteristics of the catchment.

### Attribution analysis of temporal and spatial variation

#### Attribution analysis of temporal variation of concentration of NH_3_-N and TP

The temporal variation characteristics of NH_3_-N in 7 catchments out of 13 catchments were mainly affected by three factors: PWA, PIA, PPI. The importance rankings of these three factors were different among catchments. The temporal variation characteristics of NH_3_-N in other catchments were significantly affected by RC, PSI, PTI, SHDI, and PGA factors (Tables 4 and 5).

**Table 4:** Values of the regression coefficients from PLSR models which describe the relationships (direction and magnitude) between influencing factors and temporal variation of NH_3_-N in each catchment

Coefficient	Number of Catchment												
	1	2	3	4	5	6	7	8	9	10	11	12	13
Constant term	21.227	/	−28.271	2.393	−11.600	6.107	7.113	/	17.348	−33.108	5.206	2.338	−18.012
RC	**0.572**	0.297	−0.046	−0.977	−0.479	0.389	0.216	−0.062	−0.098	−0.100	0.253	−2.635	−0.296
pH	−0.063	−0.009	−2.817	0.008	−0.763	0.026	0.012	−0.302	−0.181	−0.926	0.097	1.800	−0.167
DO	0.003	0.089	0.727	0.298	0.164	0.043	0.124	0.176	0.243	−0.025	0.234	−0.098	−0.034
COD_Mn_	0.077	0.077	−0.070	0.142	0.204	−0.033	0.253	0.140	0.122	−0.259	−0.019	0.367	0.095
PFA	−0.059	0.010	0.222	−0.052	−0.035	−0.035	−0.004	−0.053	−0.117	0.087	−0.049	−0.113	−0.144
PGA	0.051	−0.020	−0.532	0.069	0.048	0.040	0.005	0.147	0.135	−0.191	0.083	0.152	**−62.298**
PWA	**−2.729**	**1371.300**	**−180.910**	−3.130	**−3.637**	**−8.377**	**−0.588**	**−2.747**	**−31.079**	**5.926**	**−6.850**	**−5.891**	**−6.244**
PCA	0.349	−0.034	−0.374	0.164	0.124	0.558	0.018	0.081	0.718	−0.159	0.104	0.481	0.127
PIA	**2.722**	0.480	**12.191**	−0.527	**−1.821**	**2.277**	−0.210	**3.755**	**7.317**	**9.423**	**−1.958**	**11.357**	−1.367
PD	0.124	0.001	0.019	−0.002	−0.002	−0.009	−0.004	−0.002	−0.008	0.002	−0.003	−0.006	−0.002
LSI	−0.001	||<0.001	0.012	−0.001	||<0.001	−0.001	−0.001	−0.001	−0.001	||<0.001	−0.001	−0.001	−0.001
COHESION	0.207	−0.006	−0.123	0.012	0.027	0.094	0.074	0.042	0.155	−0.085	0.020	0.069	0.028
SHDI	−0.019	0.015	0.288	−0.052	−0.068	−0.037	**−0.274**	−0.046	−0.117	0.093	−0.047	−0.124	−0.191
Average Slope	||<0.001	/	||<0.001	||<0.001	||<0.001	||<0.001	||<0.001	/	||<0.001	||<0.001	||<0.001	||<0.001	||<0.001
HI	||<0.001	/	||<0.001	||<0.001	||<0.001	||<0.001	||<0.001	/	||<0.001	||<0.001	||<0.001	||<0.001	||<0.001
Pop_ Den	||<0.001	/	0.077	||<0.001	−0.060	−0.019	||<0.001	−0.013	||<0.001	−0.138	||<0.001	−0.042	−0.001
Liv_Den	0.005	||<0.001	0.013	0.003	0.002	||<0.001	||<0.001	||<0.001	||<0.001	||<0.001	||<0.001	||<0.001	||<0.001
GRDP	||<0.001	||<0.001	−0.001	||<0.001	||<0.001	||<0.001	||<0.001	||<0.001	||<0.001	||<0.001	||<0.001	||<0.001	||<0.001
PPI	0.004	**−1.148**	**−60.850**	**−33.721**	**−33.028**	−0.092	**−0.847**	**−5.346**	**−20.885**	**−9.443**	**−30.571**	**57.005**	**3.092**
PSI	−0.059	−0.221	5.251	**5.785**	1.765	−0.227	0.023	0.579	0.560	0.283	0.392	−0.183	−1.117
PTI	0.090	**2.131**	−4.575	**−5.520**	−1.710	**0.587**	0.151	−0.638	−0.556	−0.027	−0.375	−0.097	−0.295

|| represents the absolute value of the coefficient. Bold font is used to identify the top three important independent variables in each catchment based on PLSR

**Table 5:** Results from the PLSR models for temporal variation of NH_3_-N in each catchment

Number of Catchment	Name	Mean concentration (mg/L)	R^2^	RMSE	Significant Factors of Influence		
1	Jintan	0.19	0.15	0.91	PWA	PIA	RC
2	Zhamalong	0.27	0.54	0.67	PWA	PTI	PPI
3	Xiaoxiaqiao	1.88	0.30	0.83	PWA	PPI	PIA
4	Wanziqiao	1.73	0.23	0.87	PPI	PSI	PTI
5	Laoyaxiakou	1.55	0.17	0.90	PPI	PWA	PIA
6	Taerqiao	0.11	0.26	0.85	PWA	P0.00IA	PTI
7	Xinningqiao-Datong	0.48	0.61	0.62	PPI	PWA	SHDI
8	Runzeqiao	0.54	0.53	0.68	PPI	PIA	PWA
9	Chaoyangqiao	0.73	0.42	0.76	PWA	PPI	PIA
10	Sanqiqiao	0.71	0.29	0.84	PPI	PIA	PWA
11	Shatangchuanqiao	0.74	0.31	0.82	PPI	PWA	PIA
12	Qiyiqiao	1.38	0.46	0.73	PPI	PIA	PWA
13	Minheqiao	0.55	0.37	0.78	PGA	PWA	PPI

The temporal variation characteristics of TP in 8 catchments out of 13 catchments were mainly affected by three factors: PWA, PIA, PPI. The importance rankings of the three factors were different in different catchments. The temporal variation characteristics of TP in other catchments were significantly affected by PSI, PTI, PCA, PGA and RC factors (Table S2, Table 6).

**Table 6:** Results from the PLSR models for temporal variation of TP in each catchment

Number of catchment	Name	Mean concentration (mg/L)	*R*^2^	RMSE	Significant factors of influence		
1	Jintan	0.02	0.34	0.81	PWA	PIA	PPI
2	Zhamalong	0.07	0.65	0.58	PWA	PTI	PIA
3	Xiaoxiaqiao	0.22	0.58	0.64	PWA	PPI	PIA
4	Wanziqiao	0.23	0.44	0.74	PPI	PTI	PSI
5	Laoyaxiakou	0.21	0.24	0.86	PPI	PWA	PIA
6	Taerqiao	0.03	0.44	0.74	PWA	PIA	PCA
7	Xinningqiao-Datong	0.14	0.91	0.29	PWA	PIA	PPI
8	Runzeqiao	0.13	0.58	0.64	PIA	PWA	PPI
9	Chaoyangqiao	0.18	0.25	0.86	PWA	PIA	RC
10	Sanqiqiao	0.16	0.29	0.84	PPI	PIA	PWA
11	Shatangchuanqiao	0.15	0.51	0.69	PPI	PWA	PIA
12	Qiyiqiao	0.17	0.46	0.73	PIA	PPI	PWA
13	Minheqiao	0.13	0.47	0.72	PGA	PPI	PTI

#### Attribution analysis of spatial variation of concentration of NH_3_-N and TP

As listed in Table 7, the spatial variation characteristics of NH_3_-N in the basin were mainly affected by PWA, PSI and HI. Besides, the spatial variation characteristics of TP in the basin were significantly influenced by PWA, PSI and PTI, as shown in Table 8.

**Table 7:** Values of the regression coefficients and other results from PLSR models which describe the relationships (direction and magnitude) between influencing factors and spatial variation of NH_3_-N of the whole basin

Influencing factors	Coefficient	*R*^2^	RMSE
Constant term	−3.843	0.98	0.15
RC	0.031		
pH	0.078		
DO	−0.266		
COD_Mn_	1.003		
PFA	0.022		
PGA	0.390		
PWA	95.193		
PCA	−0.399		
PIA	0.307		
PD	0.090		
LSI	0.006		
COHESION	−0.068		
SHDI	0.168		
Average Slope	−0.001		
HI	1.298		
Pop_ Den	−0.001		
Liv_Den	||<0.001		
GRDP	||<0.001		
PPI	−1.017		
PSI	−1.445		
PTI	1.054		

|| represents the absolute value of the coefficient

**Table 8:** Values of the regression coefficients and other results from PLSR models which describe the relationships (direction and magnitude) between influencing factors and spatial variation of TP of the whole basin

Influencing factors	Coefficient	*R*^2^	RMSE
Constant term	–2.596	0.99	0.07
RC	–0.096		
pH	–0.045		
DO	–0.037		
COD_Mn_	0.080		
PFA	0.111		
PGA	–0.017		
PWA	–3.895		
PCA	0.028		
PIA	0.030		
PD	0.007		
LSI	||<0.001		
COHESION	–0.023		
SHDI	0.018		
Average Slope	–0.001		
HI	0.053		
Pop_ Den	||<0.001		
Liv_Den	||<0.001		
GRDP	||<0.001		
PPI	–0.113(0)		
PSI			–0.153
PTI			0.113(2)

|| represents the absolute value of the coefficient

## Discussion

### Analysis of main controlling factors of temporal variation of NH_3_-N and TP

The temporal variation of NH_3_-N and TP in Xiaoxiaqiao, Runzeqiao, Sanqiqiao, Shatangchuanqiao, Qiyiqiao, Laoyaxiakou catchment in Huangshui Basin were mainly influenced by factors of PWA, PIA, and PPI. These catchment areas are located in the middle reaches of Huangshui River except Laoyaxiakou. The average concentration of NH_3_-N and TP in this kind of catchment area was relatively high, which may be related to the higher population density (greater than 123 p/km^2^), which was similar to the relevant studies (Alvarez-Cobelas et al., 2008; Wang et al., 2019; Cui et al., 2020). Two thirds of the abovementioned catchments were found with relatively high value of PIA (more than 0.05), and low values of the elevations. The significant influences of PWA and PIA on the temporal change of NH_3_-N and TP in these catchments reflected that the land use type was the main factor affecting the NH_3_-N and TP concentration in the middle reaches of the Huangshui watershed. Because land use types can conceivably affect the runoff processes which carry anthropogenic substances such as nutrients into rivers; therefore, certain land use types can influence on water contaminant concentrations (Zhou et al., 2012).

Pellerin et al. (2004) found that the nitrogen export through streams may be related with the ratio of wetland areas in the catchments, which promotes nitrogen retention and volatilization. Li et al. (2020) concluded that the ratio of urban areas, and the ratio of forest areas were major influential indicators that affected TN and TP in river water. Jabbar and Grote (2019) hold the view that water quality parameters P were also correlated with the percentage of urban land. Similarly, our research found that the temporal variations of ammonia nitrogen and total phosphorus were significantly influenced by PWA and PIA. The PPI of Xiaoxiaqiao and Sanqiqiao catchment in the upper reaches were higher (greater than 0.17) and the PPI of the remaining catchments were all less than 0.09. It was shown that PPI with too high or low values may make it the main controlling factor for the temporal change of NH_3_-N and TP.

The temporal change of TP in Xinningqiao-Datong catchment was significantly affected by PPI and PIA, which may be related to the higher PPI (close to 0.16) and middle PIA (about 0.06). Moreover, the temporal variation of NH_3_-N in Chaoyangqiao catchment was also mainly affected by three factors: PWA, PIA, and PPI. While the temporal change of TP was affected by RC obviously, which may be related to the low degree of human development and the distribution of temperate deciduous broad-leaved forest in the northwest of the catchment.

The temporal change of NH_3_-N in Jintan catchment was significantly affected by PWA, PIA, and RC. The Jintan catchment was located in the upper reaches of the Huangshui River with low average concentration of NH_3_-N. It may due to its lowest population density and livestock density among all the catchments. Meanwhile, the land use type of this catchment was mainly grassland and woodland (>0.85), and the impervious ground area only accounted for a small proportion (<0.01). This is in accord with that agriculture land and urban land were negatively correlated with surface water quality, whereas forested land, water area, and grassland were positively correlated with water quality (Chen et al., 2016; Lee et al., 2020; Xu et al., 2019a). The PPI was relatively high (>0.09), and the secondary industry was underdeveloped (PSI was approximately equal 0.37). In addition, the temporal change of NH_3_-N was obviously affected by the runoff coefficient, which may be closely related to the low degree of socioeconomic development in this catchment. The temporal change of TP in Jintan catchment was significantly affected by PWA, PIA and PPI, followed by PCA, COHESION and RC, which indicated that agricultural non-point source pollution in the catchment may have a relatively large contribution to the total phosphorus load.

The temporal variations of NH_3_-N and TP in Zhamalong and Wanziqiao catchments were significantly affected by PTI, of which Wanziqiao catchment was also significantly affected by PSI. This may be related to the small value of average PTI of Zhamalong catchment (<0.3), and the larger value of the sum of PTI and PSI in Wanziqiao catchment (>0.9). The results were similar with (Xu et al., 2019b), which found wastewater from sewage outfalls was the largest contributor (26.2%) to AN pollution in dry seasons in Huaihe River basin.

The temporal change of NH_3_-N in the Taerqiao catchment and the temporal change of TP in the Minheqiao catchment were both significantly affected by PTI, which indicated that socioeconomic activities, especially NH_3_-N and TP discharge from the tertiary industry, have become a serious problem for the entire Huangshui basin. Meanwhile, the temporal change of TP in the Taerqiao catchment was obviously affected by PCA, which may be related to the lower average PCA (close to 0.16) in the catchment. The temporal variation of TP in the Minheqiao catchment was obviously affected by PGA which maybe related with the lower mean value of PGA.

### Analysis of main controlling factors of spatial variation of NH_3_-N and TP

**Fig. 9:**
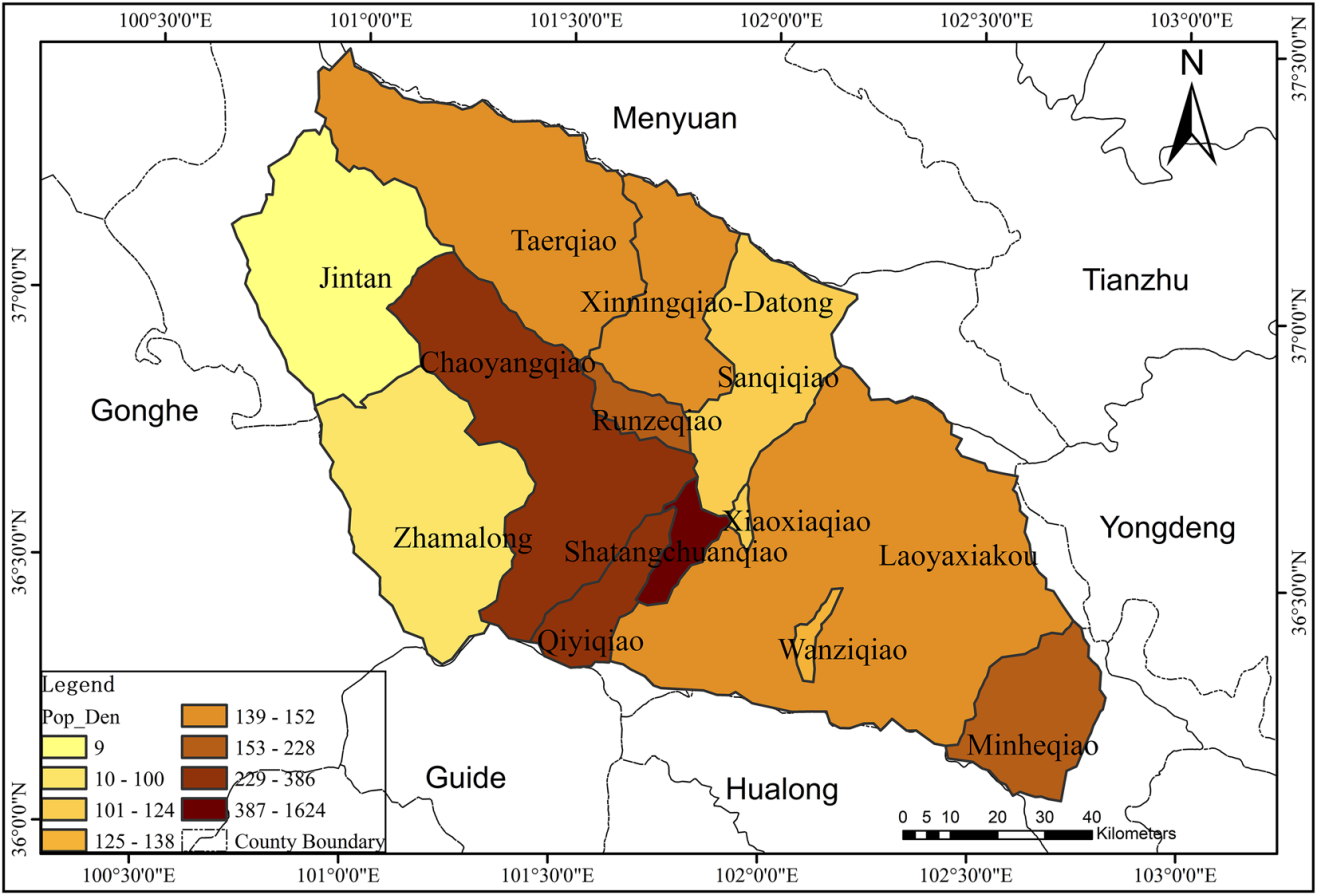
Distribution map of population density in each catchment of Huangshui River basin

**Fig. 10:**
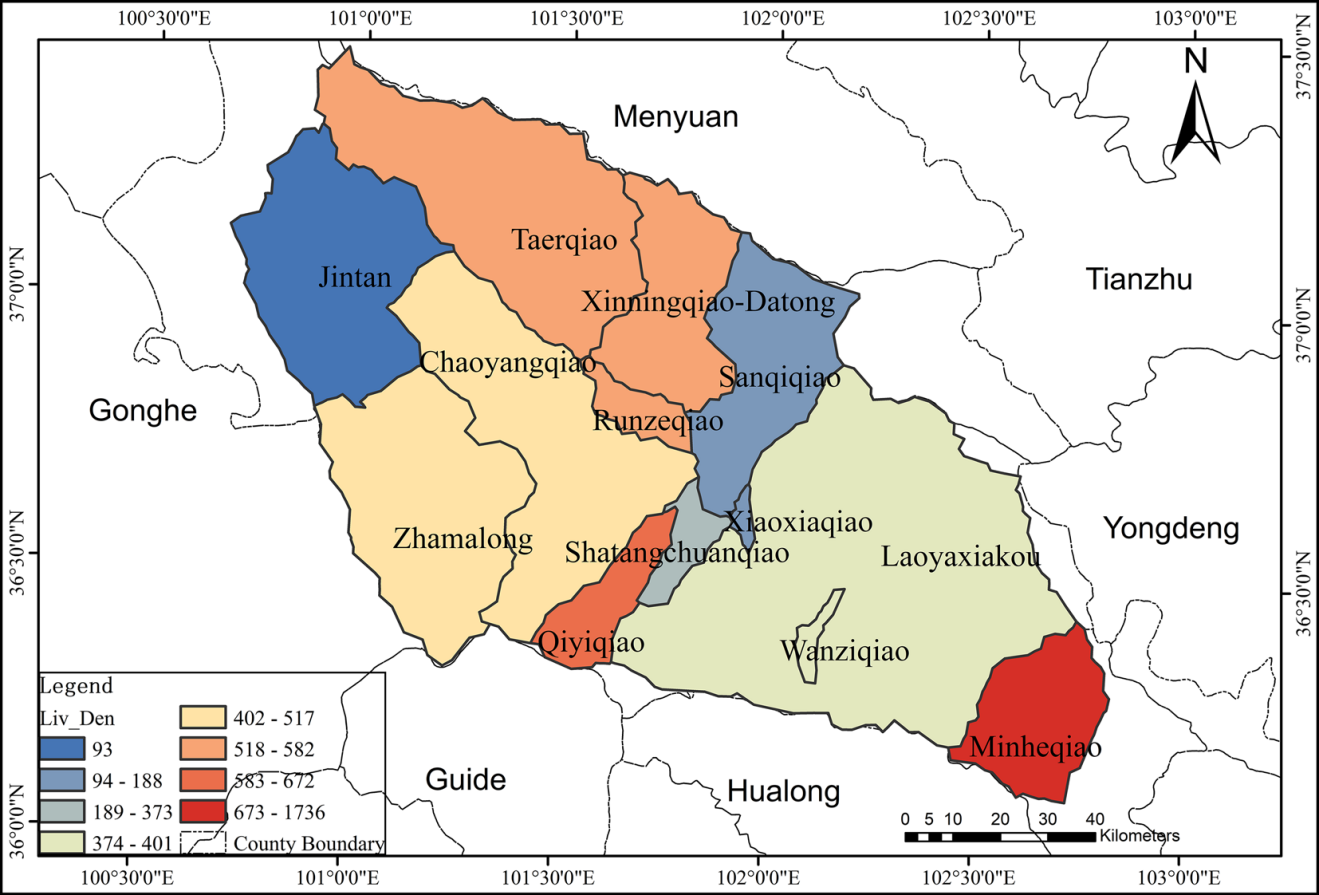
Distribution map of livestock density in each catchment of Huangshui River basin

**Fig. 11:**
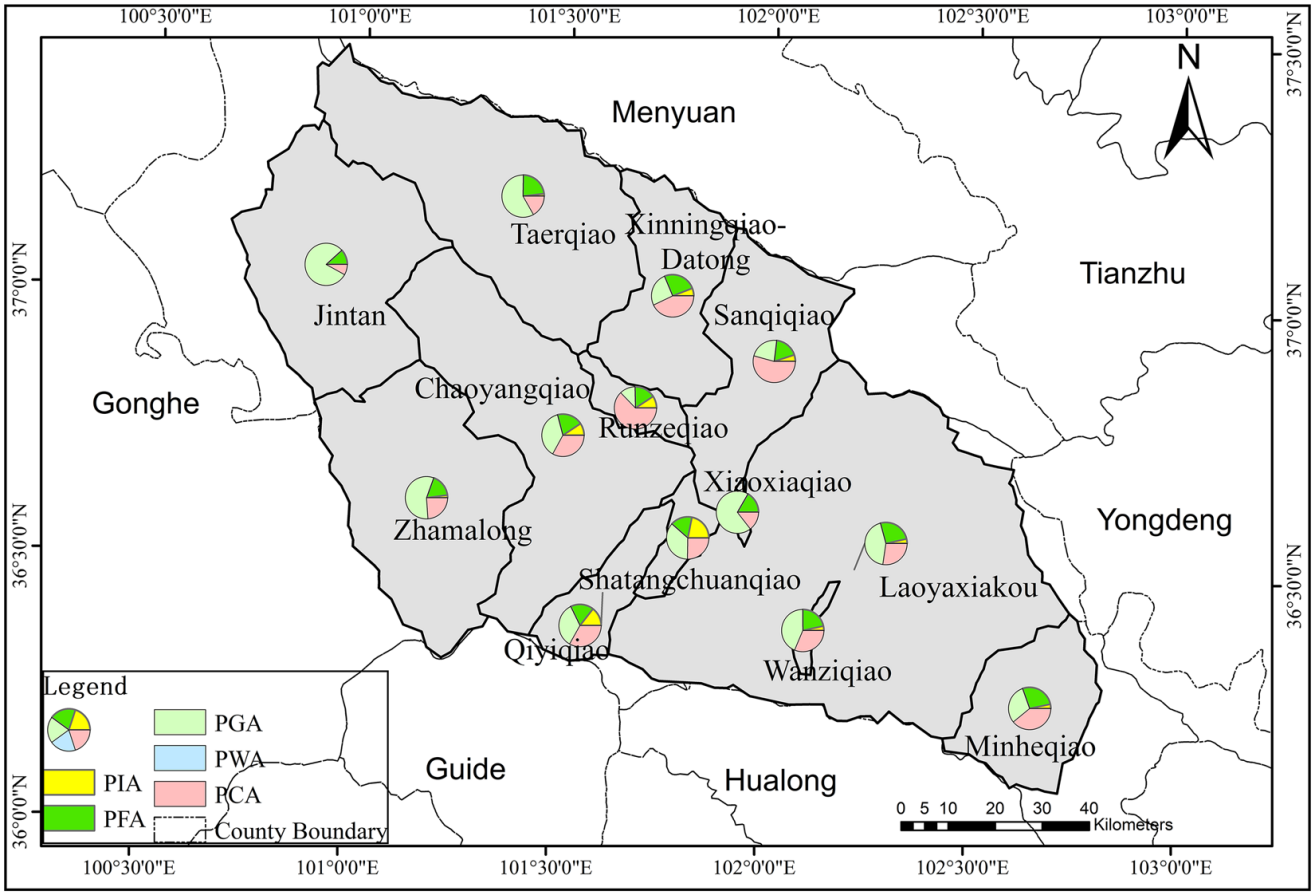
Proportion of area of main land use types in each catchment of Huangshui River basin

There was a certain correlation between the spatial variation and the temporal change of NH_3_-N concentration. The temporal changes of NH_3_-N in 12 catchments out of 13 catchments were significantly influenced by PWA. Therefore, the spatial variability of NH_3_-N concentration was also affected by PWA to some extent. The natural attributes of the watershed (topography, land use) are closely related to the factor HI. According to Yu et al. (2020), the large-scale vegetation restoration engineering in Loess Plateau since 1990s converted slope farmlands into forests or grasslands. This indicated that the land use change can be reflected by HD of the catchments to some degree. Considering the distinct influences of land use types on water contaminant concentrations, the relevant factor HI could be the significant factor for spatial variation of NH_3_-N. The intensity of socioeconomic development and industrial structure layout of a basin both correlated with PSI, which contributes a lot to point sources of ammonia nitrogen pollution discharge. According to incomplete statistics, more than 70% of the industrial and mining enterprises in Qinghai Province are distributed in the Huangshui River basin, which is called the “Mother River” of Qinghai. Therefore, PSI in the Huangshui River basin undoubtedly contributes significantly to the spatial variation of ammonia nitrogen in this basin, which was similar with the conclusion of Xu et al. (2019a). The spatial variability of NH_3_-N concentration was mainly affected by the three factors: PWA, HI and PSI, which were similar to the findings of Ai et al. (2015) and Rattan et al. (2017) (Figs. 9, 10, and 11).

**Fig. 12:**
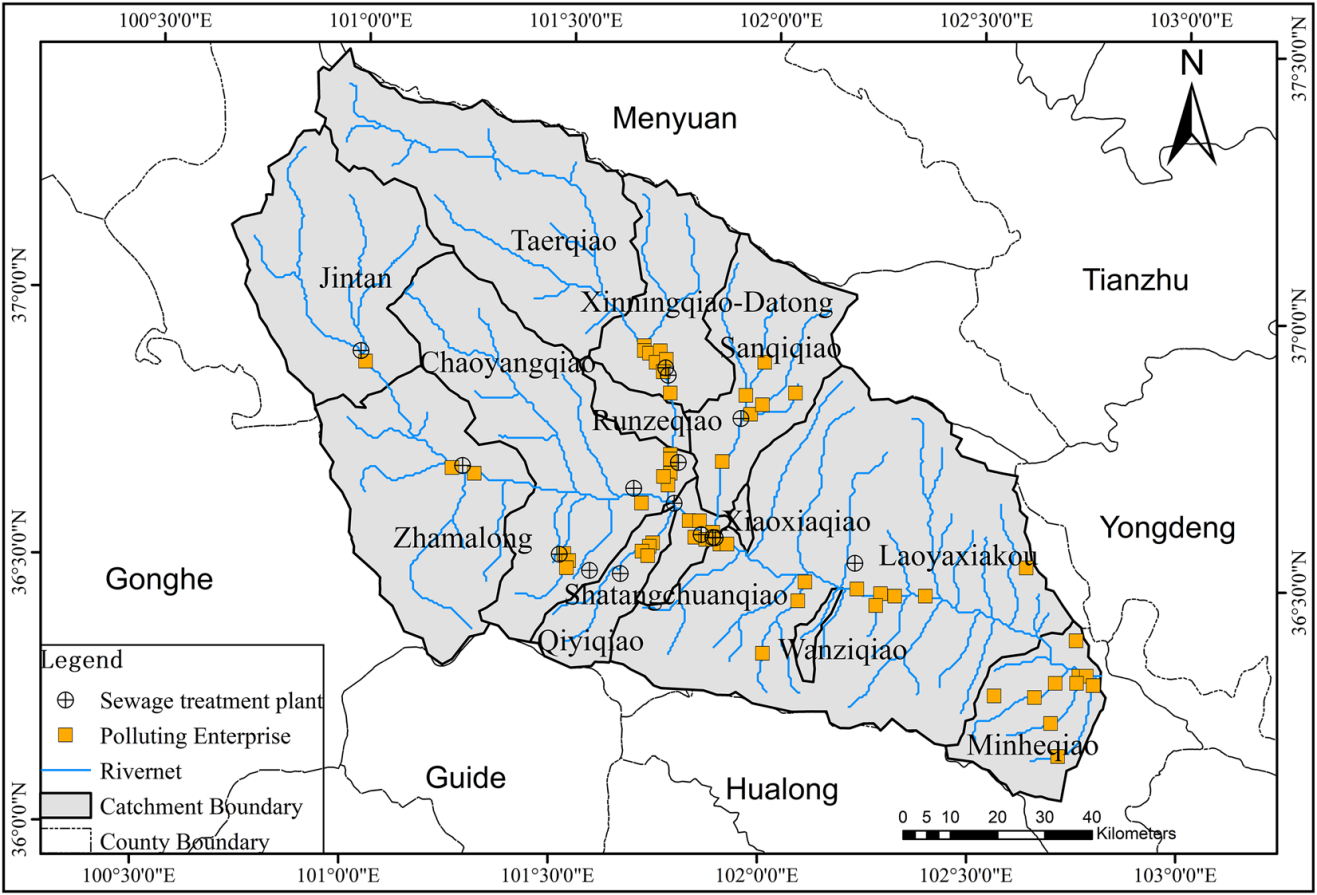
A preliminary statistics of sewage treatment plants and polluting enterprise distribution in Huangshui River basin

The certain correlation also existed between the spatial variation and the temporal change of TP concentration. The temporal change of TP in 11 catchments out of 13 catchments was significantly affected by PWA. Thus, the spatial variability of TP concentration was also affected by PWA obviously. Similar to the research of Zhou (2012), the sources of total phosphorus pollution load in Huangshui River Basin from 1997 to 2000 were mainly domestic, industrial wastewater, and fertilizer use, followed by livestock and poultry breeding. It showed that the total phosphorus pollution load of the secondary and tertiary industries was higher than that of the primary industry. Moreover, most of the sewage treatment plants and polluting enterprises in the basin are located along the river or close to the river net (Fig. 12). This type of discharge belongs to point source pollution, which may explain that the spatial variation of total phosphorus concentration was significantly affected by PSI and PTI. It also indicated that socioeconomic activities, especially NH_3_-N and TP discharge from the secondary and tertiary industry, have become a serious problem for the entire basin.

## Conclusions

In this study, 4 out of 7 catchments in the middle reaches of the Huangshui River basin were found to have a larger number of mutations of NH_3_-N (>4) through the M–K test. The catchments in the upstream and downstream areas except Jintan and Taerqiao had fewer mutations of NH_3_-N and TP. The cluster analysis results of NH_3_-N and TP of all catchments were similar according to hierarchical agglomerative cluster analysis. The concentration of NH_3_-N and TP in the upstream and at the outlet of the basin has a higher possibility to meet the Class III standards for surface water bodies in GB3838-2002) (CSEPB, 2002).

This study showed that although the Huangshui River Basin was located in the upper reaches of the Yellow River, the influences of rainfall-runoff relationship on spatiotemporal changes of NH_3_-N and TP in its sub-basins were limited. Only the temporal change of NH_3_-N in Jintan catchment in the upstream area was significantly affected by runoff coefficient. Based on PLSR, land use types and socioeconomic development were the two main categories of factors which affect the temporal changes of NH_3_-N and TP in Huangshui River basin, of which PWA, PIA, and PPI were the top three independent factors for most catchments in the middle reaches. The temporal variations of NH_3_-N and TP in the large majority (12/13 of total catchments for NH_3_-N, 11/13 for TP) of all catchments were significantly affected by PWA. In the middle reaches of the Huangshui River basin, the temporal variations of NH_3_-N and TP in most catchments (6/7 of total catchments for NH_3_-N, 5/7 of total catchments for TP) were also significantly affected by PIA and PPI. The temporal variation characteristics of TP in other catchments were significantly affected by PCA and PGA factors. The spatial variation of NH_3_-N was mainly affected by PWA, HI, and PSI. The spatial variation of TP was significantly affected by PWA, PSI, and PTI. The conclusions of this study may provide a reference for the control and management of nitrogen and phosphorus pollution in similar upstream river basins.

## Supplementary Information

Below is the link to the electronic supplementary material.

Supplementary file1 (DOCX 16 KB)

Supplementary file2 (DOCX 21 KB)

## References

[CR1] Ai L, Shi ZH, Yin W, Huang X (2015). Spatial and seasonal patterns in stream water contamination across mountainous watersheds: Linkage with landscape characteristics. Journal of Hydrology.

[CR2] Al-Murairi N, Abahussain A, El-Bettay A (2014). Spatial and temporal characterizations of water quality in Kuwait Bay. Marine Pollution Bulletin.

[CR3] Alvarez-Cobelas M, Angeler DG, Sanchez-Carrillo S (2008). Export of nitrogen from catchments: A worldwide analysis. Environmental Pollution.

[CR4] Buendia C, Bussi G, Tuset J, Vericat D, Sabater S, Palau A (2016). Effects of afforestation on runoff and sediment load in an upland Mediterranean catchment. Science of the Total Environment.

[CR5] Carrascal LM, Galvan I, Gordo O (2009). Partial least squares regression as an alternative to current regression methods used in ecology. Oikos.

[CR6] Chen Q, Mei K, Dahlgren RA, Wang T, Gong J, Zhang MH (2016). Impacts of land use and population density on seasonal surface water quality using a modified geographically weighted regression. Science of the Total Environment.

[CR7] CSEPB (Chinese State Environment Protection Bureau). (2002). Environmental Quality Standards for Surface Water (GB3838–2002).

[CR8] Cui X, Huang CZ, Wu JP, Liu XH, Hong YG (2020). Temporal and spatial variations of net anthropogenic nitrogen inputs (NANI) in the Pearl River Basin of China from 1986 to 2015. PLoS One.

[CR9] Dupas R, Minaudo C, Gruau G, Ruiz L, Gascuel-Odoux C (2018). Multidecadal trajectory of riverine nitrogen and phosphorus dynamics in rural catchments. Water Resources Research.

[CR10] Fathian F, Dehghan Z, Bazrkar MH, Eslamian S (2016). Trends in hydrological and climatic variables affected by four variations of the Mann-Kendall approach in Urmia Lake basin. Iran. Hydrological Sciences Journal-Journal Des Sciences Hydrologiques.

[CR11] Guo D, Lintern A, Webb JA, Ryu D, Liu S, Bende-Michl U (2019). Key factors affecting temporal variability in stream water quality. Water Resources Research.

[CR12] Han Q, Tong RZ, Sun WC, Zhao Y, Yu JS, Wang GQ (2020). Anthropogenic influences on the water quality of the Baiyangdian Lake in North China over the last decade. Science of the Total Environment.

[CR13] He S, Qin TL, Liu F, Liu SS, Dong BQ, Wang JW (2019). Effects of slope ecological restoration on runoff and its response to climate change. International Journal of Environmental Research and Public Health.

[CR14] Hobbie SE, Finlay JC, Janke BD, Nidzgorski DA, Millet DB, Baker LA (2017). Contrasting nitrogen and phosphorus budgets in urban watersheds and implications for managing urban water pollution. Proceedings of the National Academy of Sciences of the United States of America.

[CR15] Huang GR, Li KM, Zeng XH, Hu HY, Ren XW (2014). Watershed non-point source pollution load accounting.

[CR16] Jabbar FK, Grote K (2019). Statistical assessment of nonpoint source pollution in agricultural watersheds in the Lower Grand River watershed, MO, USA. Environmental Science and Pollution Research.

[CR17] Lee JW, Lee SW, An KJ, Hwang SJ, Kim NY (2020). An estimated structural equation model to assess the effects of land use on water quality and benthic macroinvertebrates in streams of the Nam-Han River System, South Korea. International Journal of Environmental Research and Public Health.

[CR18] Li CL, Filho WL, Wang J, Yin J, Fedoruk M, Bao G (2018). An assessment of the impacts of climate extremes on the vegetation in Mongolian Plateau: Using a scenarios-based analysis to support regional adaptation and mitigation options. Ecological Indicators.

[CR19] Li CW, Zhang HY, Hao YH, Zhang M (2020). Characterizing the heterogeneous correlations between the landscape patterns and seasonal variations of total nitrogen and total phosphorus in a peri-urban watershed. Environmental Science and Pollution Research.

[CR20] Liu F, Qin TL, Yan DH, Wang Y, Dong BQ, Wang JW (2020). Classification of instream ecological water demand and crucial values in a semi-arid river basin. Science of the Total Environment.

[CR21] Liu XC, Beusen AHW, Van Beek LPH, Mogollon JM, Ran XB, Bouwman AF (2018). Exploring spatiotemporal changes of the Yangtze River (Changjiang) nitrogen and phosphorus sources, retention and export to the East China Sea and Yellow Sea. Water Research.

[CR22] Luedeling E, Gassner A (2012). Partial least squares regression for analyzing walnut phenology in California. Agricultural and Forest Meteorology.

[CR23] Luo K, Hu XB, He Q, Wu ZS, Cheng H, Hu ZL (2017). Using multivariate techniques to assess the effects of urbanization on surface water quality: A case study in the Liangjiang New Area. China. Environmental Monitoring and Assessment.

[CR24] Mayora G, Schneider B, Rossi A (2018). Turbidity and dissolved organic matter as significant predictors of spatio-temporal dynamics of phosphorus in a large river-floodplain system. River Research and Applications.

[CR25] McGarigal, K., Cushman, S., & Ene, E. (2015). FRAGSTATS v4: Spatial Pattern Analysis Program for Categorical Maps. Computer Software Program Produced by the Authors at the University of Massachusetts, Amherst. https://www.umass.edu/landeco/research/fragstats/downloads/fragstats_downloads.html Accessed 27 September 2020.

[CR26] Mellander P-E, Jordan P, Shore M, Melland AR, Shortle G (2015). Flow paths and phosphorus transfer pathways in two agricultural streams with contrasting flow controls. Hydrological Processes.

[CR27] Min M, Lee WS (2005). Determination of significant wavelengths and prediction of nitrogen content for citrus. Transactions of the ASAE.

[CR28] Ministry of Ecology and Environment of the People’s Republic of China. (2018). *China Environmental Status Bulletin.* http://www.mee.gov.cn/hjzl/sthjzk/zghjzkgb/ Accessed 26 September 2020.

[CR29] Nielsen A, Trolle D, Søndergaard M, Lauridsen TL, Bjerring R, Olesen JE (2012). Watershed land use effects on lake water quality in Denmark. Ecological Applications.

[CR30] Nobre RLG, Caliman A, Cabral CR, Araujo FD, Guerin J, Dantas FDC (2020). Precipitation, landscape properties and land use interactively affect water quality of tropical freshwaters. Science of the Total Environment.

[CR31] Oliveira, M. R., Branco, J. A., Croux, C., & Filzmoser, P. (2004). Robust redundancy analysis by alternating regression. International Conference on Robust Statistics (ICORS 2003), Antwerp, Belgium, 13–18 Jul 2003.

[CR32] Outram FN, Cooper RJ, Sunnenberg G, Hiscock KM, Lovett AA (2016). Antecedent conditions, hydrological connectivity and anthropogenic inputs: Factors affecting nitrate and phosphorus transfers to agricultural headwater streams. Science of the Total Environment.

[CR33] Pathak D, Whitehead PG, Futter MN, Sinha R (2018). Water quality assessment and catchment-scale nutrient flux modeling in the Ramganga River Basin in north India: An application of INCA model. Science of the Total Environment.

[CR34] Pellerin, B. A., Wollheim, W. M., Hopkinson, C. S., McDowell, W. H., Williams, M. R., Vörösmarty, C. J., & Daley, M. L. (2004). Role of wetlands and developed land use on dissolved organic nitrogen concentrations and DON/TDN in northeastern US rivers and streams. *Limnology and Oceanography*, *49*(4), 910–918.

[CR35] Pennino M, Kaushal SS, Mayer PM, Utz RM, Cooper CA (2016). Stream restoration and sewers impact sources and fluxes of water, carbon, and nutrients in urban watersheds. Hydrology and Earth System Sciences.

[CR36] Rattan KJ, Corriveau JC, Brua RB, Culp JM, Yates AG, Chambers PA (2017). Quantifying seasonal variation in total phosphorus and nitrogen from prairie streams in the Red River Basin, Manitoba Canada. Science of the Total Environment.

[CR37] Razmkhah H, Abrishmchi A, Torkian A (2010). Evaluation of spatial and temporal variation in water quality by pattern recognition techniques: A case study on Jajrood River (Tehran, Iran). Journal of Environmental Management.

[CR38] Reichwaldt ES, Ghadouani A (2016). Can mussels be used as sentinel organisms for characterization of pollution in urban water systems?. Hydrology and Earth System Sciences.

[CR39] Rodrigues V, Estrany J, Ranzini M, de Cicco V, Martín-Benito JMT, Hedo J (2018). Effects of land use and seasonality on stream water quality in a small tropical catchment: the headwater of Córrego Água Limpa, São Paulo (Brazil). Science of the Total Environment.

[CR40] Shi W, Xia J, Zhang X (2016). Influences of anthropogenic activities and topography on water quality in the highly regulated Huai River basin. China. Environmental Science and Pollution Research.

[CR41] Srinivas R, Singh AP, Dhadse K, Garg C (2020). An evidence based integrated watershed modelling system to assess the impact of non-point source pollution in the riverine ecosystem. Journal of Cleaner Production.

[CR43] Wang HT, Ma B, Tang SZ, Chen ZX, Li L, Wang JL (2019). Evaluation on the Status Quo of Water Environmental for the Upper and Middle Reaches of the Yarlung Zangbo River in the Low Water Period. Fresenius Environmental Bulletin.

[CR44] Wold S, van der Waterbeemd H (1995). PLS for multivariate linear modeling. Chemometric Methods in Molecular Design: Methods and Principles in Medicinal Chemistry.

[CR45] Xia XH, Zhang SB, Li SL, Zhang LW, Wang GQ, Zhang L (2018). The cycle of nitrogen in river systems: sources, transformation, and flux. Environmental Science-Processes & Impacts.

[CR46] Xiao R, Wang GF, Zhang QW, Zhang ZH (2016). Multi-scale analysis of relationship between landscape pattern and urban river water quality in different seasons. Scientific Reports.

[CR47] Xu GC, Li P, Lu KX, Zhan TT, Zhang JX, Ren ZP (2019). Seasonal changes in water quality and its main influencing factors in the Dan River basin. CATENA.

[CR48] Xu, J., Jin, G. Q., Tang, H. W., Mo, Y. M., Wang, Y. G., & Li, L. (2019b). Response of water quality to land use and sewage outfalls in different seasons. *Science of the Total Environment*, 696, UNSP 134014.

[CR49] Yu Y, Wei W, Chen LD, Jia FY, Yang L, Zhang HD (2015). Responses of vertical soil moisture to rainfall pulses and land uses in a typical loess hilly area. China. Solid Earth.

[CR50] Yu Y, Zhao WW, Martinez-Murillo JF, Pereira P (2020). Loess Plateau: from degradation to restoration. Science of the Total Environment.

[CR51] Zhang J, Li DP, Gao P, Tao Y, Wang XM, He XH (2011). Analysis of water quality factors influencing the speciation of inorganic nitrogen using GRA. Journal of Environmental Biology.

[CR52] Zhao Y, Karypis G (2005). Hierarchical clustering algorithms for document datasets. Data Mining and Knowledge Discovery.

[CR53] Zhou T, Wu JG, Peng SL (2012). Assessing the effects of landscape pattern on river water quality at multiple scales: a case study of the Dongjiang River watershed, China. Ecological Indicators.

[CR54] Zhou, W. (2012). Analysis of nutrient sources in Huangshui River Basin based on SWAT and nutrient pollution control measures. Master's thesis, Capital Normal University, Beijing, China.

[CR55] Zhou, X. W. (2007). The study on the grey relational degree and its application. Master's thesis, Jilin University, Changchun, China.

