# Supplementary material for: Spatiotemporal variation of nitrogen and phosphorus and its main influencing factors in Huangshui River basin
Source: Environ Monit Assess. 2021 Apr 23;193(5):292. doi: 10.1007/s10661-021-09067-1 (PMC8065014; doi:10.1007/s10661-021-09067-1)
Supplement: Supplementary file 1 — Supplementary file1 (DOCX 16 KB) [file 10661_2021_9067_MOESM1_ESM.docx]

**Table S1** Summary of the numbers of mutations of NH_3_-N and TP in each catchment.

| *Number of catchment* | *Name* | *Section of River* | *Number of mutation of NH_3_-N* | *Number of mutation of TP* |
| --- | --- | --- | --- | --- |
| 1 | Jintan | Upper reaches | 4 | 4 |
| 2 | Zhamalong | Upper reaches | 1 | 1 |
| 3 | Xiaoxiaqiao | Middle reaches | 2 | 1 |
| 4 | Wanziqiao | Middle reaches | 1 | 1 |
| 5 | Laoyaxiakou | Lower reaches | 3 | 1 |
| 6 | Taerqiao | Upper reaches | 2 | 3 |
| 7 | Xinningqiao-Datong | Upper reaches | 1 | 1 |
| 8 | Runzeqiao | Middle reaches | 5 | 3 |
| 9 | Chaoyangqiao | Middle reaches | 5 | 7 |
| 10 | Sanqiqiao | Middle reaches | 4 | 1 |
| 11 | Shatangchuanqiao | Middle reaches | 6 | 1 |
| 12 | Qiyiqiao | Middle reaches | 1 | 1 |
| 13 | Minheqiao | Lower reaches | 3 | 1 |
