# Supplementary material for: Spatiotemporal variation of nitrogen and phosphorus and its main influencing factors in Huangshui River basin
Source: Environ Monit Assess. 2021 Apr 23;193(5):292. doi: 10.1007/s10661-021-09067-1 (PMC8065014; doi:10.1007/s10661-021-09067-1)
Supplement: Supplementary file 2 — Supplementary file2 (DOCX 21 KB) [file 10661_2021_9067_MOESM2_ESM.docx]

**Table S2** Values of the regression coefficients from PLSR models which describe the relationships (direction and magnitude) between influencing factors and temporal variation of TP in each catchment.

| *Coefficient* | *Number of Catchment* | | | | | | | | | | | | |
| --- | --- | --- | --- | --- | --- | --- | --- | --- | --- | --- | --- | --- | --- |
|  | *1* | *2* | *3* | *4* | *5* | *6* | *7* | *8* | *9* | *10* | *11* | *12* | *13* |
| Constant term | -3.600 | / | -1.500 | 0.999 | -1.740 | 2.068 | -70.749 | / | -0.855 | 2.108 | -0.051 | 0.501 | 0.407 |
| RC | 0.027 | 0.184 | -0.123 | -0.171 | -0.106 | 0.047 | -0.017 | -0.054 | -0.257 | -0.146 | 0.031 | -0.159 | -0.122 |
| pH | -0.008 | 0.043 | -0.075 | 0.008 | 0.096 | -0.017 | -0.035 | -0.026 | -0.145 | -0.008 | -0.115 | 0.078 | -0.058 |
| DO | -0.004 | -0.004 | 0.025 | 0.011 | -0.006 | -0.001 | 0.049 | 0.024 | 0.041 | 0.012 | -0.002 | -0.033 | -0.003 |
| COD_Mn_ | 0.003 | 0.023 | 0.015 | 0.022 | 0.006 | 0.007 | 0.064 | 0.016 | -0.005 | -0.014 | -0.002 | 0.049 | -0.007 |
| PFA | 0.010 | -0.036 | 0.018 | 0.002 | -0.010 | -0.009 | -0.367 | -0.017 | -0.005 | -0.006 | -0.029 | -0.018 | -0.015 |
| PGA | -0.009 | 0.073 | -0.042 | -0.003 | 0.013 | 0.010 | 0.466 | 0.047 | 0.006 | 0.012 | 0.050 | 0.024 | -6.634 |
| PWA | 0.479 | 272.040 | -14.301 | 0.119 | -1.002 | -2.071 | -51.545 | -0.871 | -1.367 | -0.384 | -4.139 | -0.919 | -0.665 |
| PCA | -0.061 | 0.127 | -0.030 | -0.006 | 0.034 | 0.138 | 1.602 | 0.026 | 0.032 | 0.010 | 0.063 | 0.075 | 0.013 |
| PIA | -0.478 | -1.777 | 0.964 | 0.020 | -0.502 | 0.563 | -18.396 | 1.191 | 0.322 | -0.611 | -1.183 | 1.772 | -0.146 |
| PD | -0.022 | -0.002 | 0.002 | \|\|<0.001 | -0.001 | -0.002 | 0.040 | -0.001 | \|\|<0.001 | \|\|<0.001 | -0.002 | -0.001 | \|\|<0.001 |
| LSI | \|\|<0.001 | \|\|<0.001 | 0.001 | \|\|<0.001 | \|\|<0.001 | \|\|<0.001 | 0.006 | \|\|<0.001 | \|\|<0.001 | \|\|<0.001 | -0.001 | \|\|<0.001 | \|\|<0.001 |
| COHESION | -0.036 | 0.023 | -0.010 | \|\|<0.001 | 0.008 | 0.023 | -0.667 | 0.013 | 0.007 | 0.005 | 0.012 | 0.011 | 0.003 |
| SHDI | 0.003 | -0.057 | 0.023 | 0.002 | -0.019 | -0.009 | 2.481 | -0.015 | -0.005 | -0.006 | -0.029 | -0.019 | -0.020 |
| Average Slope | \|\|<0.001 | / | \|\|<0.001 | \|\|<0.001 | \|\|<0.001 | \|\|<0.001 | \|\|<0.001 | / | \|\|<0.001 | \|\|<0.001 | \|\|<0.001 | \|\|<0.001 | \|\|<0.001 |
| HI | \|\|<0.001 | / | \|\|<0.001 | \|\|<0.001 | \|\|<0.001 | \|\|<0.001 | \|\|<0.001 | / | \|\|<0.001 | \|\|<0.001 | \|\|<0.001 | \|\|<0.001 | \|\|<0.001 |
| Pop_ Den | \|\|<0.001 | / | 0.006 | \|\|<0.001 | -0.022 | -0.001 | -0.048 | -0.004 | -0.001 | 0.014 | \|\|<0.001 | -0.003 | 0.013 |
| Liv_Den | 0.001 | \|\|<0.001 | 0.001 | \|\|<0.001 | \|\|<0.001 | \|\|<0.001 | \|\|<0.001 | \|\|<0.001 | \|\|<0.001 | \|\|<0.001 | \|\|<0.001 | \|\|<0.001 | \|\|<0.001 |
| GRDP | \|\|<0.001 | \|\|<0.001 | -0.001 | \|\|<0.001 | \|\|<0.001 | \|\|<0.001 | \|\|<0.001 | \|\|<0.001 | \|\|<0.001 | \|\|<0.001 | \|\|<0.001 | \|\|<0.001 | \|\|<0.001 |
| PPI | -0.083 | -1.267 | -3.597 | 11.724 | 2.263 | -0.024 | -4.834 | -0.835 | -0.173 | 0.776 | -10.320 | 1.202 | 1.716 |
| PSI | 0.012 | -0.156 | 0.531 | 0.597 | 0.152 | -0.021 | 1.061 | 0.115 | 0.116 | 0.108 | 0.169 | 0.093 | -0.485 |
| PTI | -0.014 | 2.056 | -0.564 | -0.708 | -0.189 | 0.060 | -1.172 | -0.132 | -0.127 | -0.171 | -0.166 | -0.113 | -0.667 |

|| represents the absolute value of the coefficient, Bold font is used to identify the top three independent variables based on PLSR.

Abbreviations of influencing factors are listed in Table 1.
